# Genetic Determinants of Wound Healing: Monogenic Disorders and Polygenic Influence

**DOI:** 10.3390/cells15010074

**Published:** 2026-01-01

**Authors:** Stephanie M. Mueller, Nalani Miller, Jasleen Gill, LaYow C. Yu, Michael Drake Pike, Dennis P. Orgill

**Affiliations:** 1 Division of Plastic and Reconstructive Surgery, Brigham and Women’s Hospital, 75 Francis Street, Boston, MA 02115, USA; smmueller@mgh.harvard.edu; 2 Harvard Medical School, 25 Shattuck Street, Boston, MA 02115, USA; jasleen_gill@hms.harvard.edu (J.G.); layow_yu@hms.harvard.edu (L.C.Y.); drake_pike@hms.harvard.edu (M.D.P.); 3 John A. Burns School of Medicine, University of Hawaiʻi at Mānoa, 651 Ilalo Street, Honolulu, HI 96813, USA; nalanilm@hawaii.edu

**Keywords:** wound healing, monogenic disease, polygenic disease, epidermolysis bullosa, Ehlers-Danlos syndrome, diabetes mellitus, obesity, sickle cell disease, leukocyte adhesion deficiency, hemophilia

## Abstract

**Highlights:**

**What are the main findings?**

- Monogenic and polygenic disorders—despite differing genetic architectures—converge on shared downstream failures in hemostasis, inflammation, cellular proliferation, and extracellular matrix remodeling.
- Monogenic conditions provide direct mechanistic insight into how discrete pathway defects impair repair, whereas polygenic diseases reveal how cumulative genetic risk and metabolic stressors overwhelm tissue regenerative capacity.

**What are the implications of the main finding?**

- Recognizing the continuum between single-gene disruptions and multifactorial genetic susceptibility enables a more mechanistically driven classification of impaired wound healing.
- By synthesizing both preclinical mechanistic studies and clinical outcome data across monogenic and polygenic conditions, this review offers a comprehensive resource for understanding genetic influences on real-world wound healing outcomes.

**Abstract:**

(1) Background: Wound healing is a highly coordinated process encompassing hemostasis, inflammation, angiogenesis, keratinocyte migration, collagen deposition, and extracellular matrix remodeling. Successful repair also requires adequate nutrient and oxygen delivery through a well-developed vascular supply. Disruption of these processes can occur through aberrations in diverse biological pathways, including extracellular matrix organization, cellular adhesions, angiogenesis, and immune regulation. (2) Methods: We reviewed mechanisms of impaired tissue repair in monogenic disorders by focusing on three categories—connective tissue, hematological/immunological, and aging-related disorders—to illustrate how single-gene defects disrupt inflammation, cellular proliferation, and matrix remodeling. Additionally, we reviewed various polygenic disorders—chronic kidney disease, diabetes mellitus, hypertension, and obesity—to contrast complex multifactorial pathologies with single-gene defects. (3) Results: This review establishes that genetic impediments, despite their distinct etiologies, monogenic and polygenic disorders share critical downstream failures in the wound healing cascade. While monogenic diseases illustrate direct causal links between specific protein deficits and repair failure, polygenic diseases demonstrate how multifactorial stressors overwhelm the body’s regenerative capacity. (4) Conclusions: This review synthesizes current evidence on both monogenic diseases and polygenic contributions to impaired wound healing. These findings highlight that genetic susceptibility is a decisive factor in the ability to restore tissue homeostasis. This underscores the profound impact of genetic background on the efficacy of hemostasis, inflammation, and remodeling.

## 1. Introduction

Wound healing is a fundamental biological process, requiring the precise coordination of inflammation, cellular proliferation, and remodeling [1,2]. Dysregulation at any phase can lead to chronic wounds or pathological fibrosis, posing a significant global clinical burden. While cellular mechanisms are well-documented, the genetic determinants of repair remain incompletely defined [2].

Repair is governed by a complex genetic network integrating growth factors (e.g., TGF-β, VEGF, FGF) and matrix metalloproteinases (MMPs) that coordinate cellular interactions [3,4]. However, these pathways are modulated by underlying genetic variation.

Monogenic disorders provide striking examples of single-gene defects profoundly altering the architecture, mechanics, and regenerative potential of tissues. These disorders function as “experiments of nature,” revealing the essential roles of specific proteins and cellular pathways. For instance, hematological and primary immunodeficiency disorders underscore the role of proper tissue oxygenation and the inflammatory response in wound repair. Premature aging disorders provide insight into the role of cell proliferation and signaling in wound healing. Lastly, connective tissue diseases reveal ECM proteins, integrins, and growth factors essential in coordinating wound repair [5].

Beyond monogenic diseases, wound healing in the general population reflects a highly polygenic architecture, shaped by the additive and interactive effects of numerous genetic loci. Genome-wide association studies (GWAS) and pathway analyses have identified variants affecting angiogenesis, immune regulation, oxidative stress, and ECM turnover. Polygenic influences underlie the variable healing capacity observed in conditions such as diabetes, peripheral artery disease, and chronic kidney disease, where cumulative genetic risk interacts with metabolic and environmental factors to influence wound healing outcomes [6,7].

While the contributions of individual gene defects and common variants to wound healing have been studied separately, their integration remains limited. Understanding the intersection of monogenic mechanisms with polygenic susceptibility is essential for developing predictive models and precision-based interventions. In this review, we synthesize current evidence on the genetic determinants of wound healing, examining both monogenic disorders that reveal fundamental biological mechanisms and polygenic influences that shape wound outcomes across populations. Together, these perspectives highlight the continuum of genetic control that governs tissue repair and illuminates emerging opportunities for translational and therapeutic innovation.

### Overview of Wound Healing Cascade

**Figure 1:**
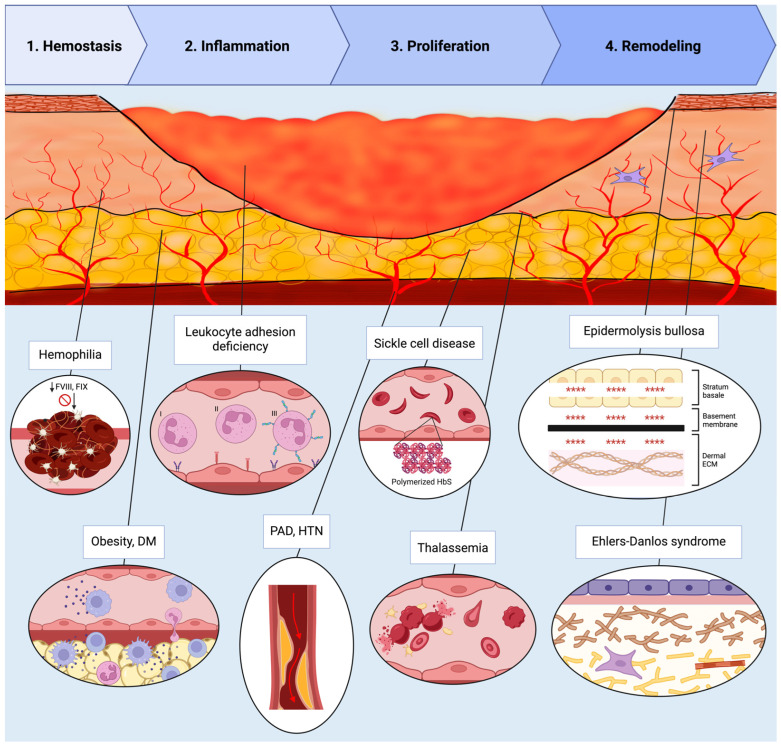
Schematic representation of the four phases of wound healing—Hemostasis, Inflammation, Proliferation, and Remodeling—illustrated within a cross-sectional wound bed. Representative monogenic and polygenic disorders impairing each phase are annotated in their corresponding regions. Descriptions are listed in order of appearance from left to right. Hemostasis: Hemophilia (FVIII and FIX deficiency) impairing fibrin clot formation. Inflammation: Obesity and diabetes mellitus promoting chronic inflammation with macrophage and lymphocyte predominance; leukocyte adhesion deficiency types I–III causing defects in neutrophil adhesion and migration. Proliferation: Peripheral arterial disease and hypertension reducing perfusion due to arterial stenosis; sickle cell disease characterized by polymerized HbS and sickled RBCs; thalassemia causing hemolysis and abnormal RBC morphology. Remodeling: Epidermolysis bullosa causing dermal–epidermal separation, as represented by the symbols “****”; Ehlers–Danlos syndrome disrupting collagen and elastin structure. Abbreviations: FVIII, factor VIII; FIX, factor IX; DM, diabetes mellitus; RBC, red blood cell; HbS, hemoglobin S; PAD, peripheral arterial disease; HTN, hypertension. Created in BioRender. Mueller, S. (2025) https://BioRender.com/t221sbh (accessed on 24 November 2025).

The restoration of tissue architecture following injury represents a remarkable feat of biological engineering, requiring the body to balance rapid closure with functional restoration. This process is governed by a delicate equilibrium of synthesis and degradation and involves hemostasis, inflammation, proliferation, and remodeling (see Figure 1).

Hemostasis begins as platelet aggregation and clotting factor activation, which halts bleeding and establishes a provisional matrix for cell migration [8]. Platelets also release growth factors, including platelet-derived growth factor (PDGF) and transforming growth factor β (TGF-β), which attract and activate macrophages and fibroblasts [3,9].

During the subsequent inflammatory phase, vasoactive and chemotactic factors recruit leukocytes; neutrophils arrive first to clear bacteria before being phagocytosed by infiltrating macrophages. These macrophages then release PDGF and vascular endothelial growth factor (VEGF) to initiate granulation. They also express cytokines, such as tumor necrosis factor α (TNF-α), interleukin-1 (IL-1), and TGF-β, that regulate the transition to the proliferative phase [3,10].

Proliferation encompasses re-epithelialization, angiogenesis, and granulation tissue formation. Keratinocytes migrate and proliferate to resurface the wound, guided by growth factors, cytokines, and MMPs [11]. Fibroblasts migrate into the wound and deposit new ECM components, while endothelial cells form new blood vessels to supply oxygen and nutrients [3,8].

Remodeling is the final phase, where the newly formed tissue undergoes maturation. Collagen fibers are reorganized and cross-linked. The wound contracts, leading to increased tensile strength. This last stage can last for months and determines the final scar outcome [11,12].

## 2. Materials and Methods

To thoroughly review the mechanisms by which monogenic disorders impair tissue repair, this paper focuses on three disease categories selected for their fundamental impact across the wound healing cascade: (1) hematological and primary immunodeficiency disorders, (2) premature aging disorders, and (3) connective tissue disorders. These categories were prioritized because they provide clear, high-impact models of how single-gene defects disrupt distinct, essential processes.

Similarly, various polygenic disorders, such as chronic kidney disease, diabetes mellitus, hypertension, obesity, and peripheral artery disease, were chosen due to their widespread clinical prevalence and their ability to act as major systemic drivers of chronic wound complications in the general population.

A targeted literature search in PubMed was conducted to identify high-yield in vitro, in vivo, and human clinical studies published between 1971 and 2025 that described genetic mechanisms relevant to wound healing for each selected disease.

## 3. Monogenic Disorders

Monogenic disorders, while individually rare, provide critical and high-fidelity insights into the fundamental pathways governing tissue repair. These conditions arise from high-penetrance mutations in a single gene, often resulting in profound and deterministic failures in cellular or structural integrity [13,14].

The following section and Table 1 describe monogenic hematological and primary immunodeficiency disorders, premature aging disorders, and connective tissue disorders, describing the genes and cellular repair mechanisms affected and detailing the preclinical and clinical evidence for impaired wound healing.

### 3.1. Hematological and Primary Immunodeficiency Disorders

Genes governing hematologic and immunologic processes play a central role in coordinating wound healing. During hemostasis, genes encoding coagulation factors (*F2*, *F5*, *F8*) and fibrinogen subunits (*FGA*, *FGB*, *FGG*) mediate clot formation, while growth factor genes (*PDGFA/B*, *TGFB1*) initiate immune recruitment and inflammation [40,41]. In the inflammatory phase, cytokine and chemokine genes (*IL1B*, *IL6*, *TNF*, *CXCL8/IL8*, *CCL2*) and adhesion molecule genes (*ITGB2I*) regulate neutrophil and macrophage activation, aided by innate immune receptors (TLR2, TLR4) and complement components (C3, C5) that facilitate pathogen clearance [42,43]. As repair progresses, genes controlling angiogenesis (*VEGFA*, *FGF2*, *EGF*) restore oxygen and nutrient delivery to the wound, both essential in the proliferative phase of wound healing [3,44].

The following section discusses the pathogenesis and wound healing issues associated with bare lymphocyte syndrome type I, hemophilia, leukocyte adhesion deficiency, sickle cell anemia, and thalassemia.

#### 3.1.1. Bare Lymphocyte Syndrome Type I (TAP-Deficiency Syndrome)

##### Gene and Protein Function

Transporter associated with antigen processing (TAP) deficiency syndrome, also known as bare lymphocyte syndrome type I (BLS I), is a rare genetic disorder characterized by significantly decreased expression of major histocompatibility complex (MHC) class I molecules [15]. It is inherited in an autosomal recessive manner and is caused by mutations in either the *TAP1* or TAP2 genes, which code for the proteins of the same name [16].

In the wild-type state, TAP1 and TAP2 proteins assemble to form the TAP complex. This complex is responsible for translocating viral antigen peptides into the endoplasmic reticulum (ER) prior to presentation on MHC class I proteins. However, mutations in the *TAP1* or *TAP2* genes cause TAP complex assembly failure. As a functional consequence, when MHC class I proteins do not have peptides to bind, they are degraded. This results in significantly fewer peptide-MHC class I complexes on the surface of cells, severely hampering CD8+ cytotoxic T-cell recognition and binding, leading to dysfunction of the adaptive immune system and vulnerability to recurrent bacterial infections [15].

##### Clinical Manifestations

BLS I is extremely rare, with only about 30 cases cited in the literature [45]. Patients tend to present in childhood with symptoms of recurrent bacterial infections, such as chronic sinusitis, nasal disease, recurrent bacterial pneumonia, postnasal-drip syndrome, otitis media, and mastoiditis. Chronic pulmonary diseases may also manifest [15].

Some patients may present with significant dermatological findings, ranging from purpura on the extremities to brownish ulcerating nodules that eventually become necrotizing granulomatous skin lesions on the extremities and the midface. This compromised skin barrier contributes to the development of recurring bacterial skin infections [15].

##### Wound Healing

Wound healing problems are clinically recognized in BLS I, but there are no direct in vitro or animal studies investigating the mechanisms or modeling these impairments in this specific syndrome. Impaired wound healing in patients with BLS I manifests as skin ulcers and necrotizing granulomatous skin lesions; however, due to the rarity of this condition, there is very little literature published that elucidates the pathophysiology behind lesion formation and impaired wound healing [15]. Though delayed healing may be explained, in part, by the increased susceptibility to infection due to the host immune system’s impaired clearance of pathogens and infected tissue [15].

Clinical reports are limited to case reports. For instance, Moins-Teisserenc et al. report five adults with BLS I due to *TAP2* mutations who developed chronic necrotizing granulomatous lesions in the skin and upper respiratory tract, recurrent infections, and skin vasculitis. The skin lesions were resistant to immunosuppressive therapy and histologically showed granulomatous inflammation with a predominance of activated natural killer (NK) cells [46]. Additionally, a case report of a 10-year-old with BLS I, confirmed with whole exome sequencing, described findings of focal subepidermal granulomatous inflammation with caseating necrosis. The patient was treated with a 60-day course of prednisolone and tacrolimus, and while some of the lesions did heal with hyperpigmented scarring, the rest of the lesions healed only partially [47].

#### 3.1.2. Hemophilia

##### Gene and Protein Function

Hemophilia is defined as a primary bleeding disorder that is inherited in an X-linked recessive pattern. Hemophilia A is characterized by a deficiency in factor VIII (FVIII), and hemophilia B is caused by dysfunctional factor IX (FIX). Over 1000 mutations to *F8* and *F9* have been identified [48].

Factor VIII and factor IX function as critical components of the intrinsic pathway of coagulation, acting as cofactors and enzymes that enable efficient thrombin generation and formation of a stable fibrin clot. A deficiency or dysfunction in either protein can impair clot formation, leading to the bleeding tendency characteristic of hemophilia [49].

##### Clinical Manifestations

Hemophilia affects all ethnic groups equally and occurs in roughly 1 in 10,000 live births, with an estimated 400,000 people worldwide living with the condition. Due to the inheritance pattern, hemophilia affects males more than females [48].

The clinical manifestations of hemophilia A and B are essentially identical and correlate with the degree of residual factor activity. Mild disease presents with bleeding only after significant trauma or surgery, whereas moderate hemophilia is characterized by bleeding after injury, dental work, or minor procedures. Severe hemophilia typically manifests in early infancy with spontaneous bleeding. The hallmark features include painful, swollen joints. This can progress to chronic hemophilic arthropathy with repeated episodes. Patients are at risk for life-threatening hemorrhage, particularly intracranial bleeding, which is a major cause of early morbidity and mortality. Additional manifestations include mucocutaneous bleeding, extensive bruising, and deep muscle hematomas [48].

##### Wound Healing

Wound healing impairments are well documented in preclinical and clinical studies.

A plasma-based in vitro model of hemophilia B demonstrated that deficient fibrin network structure compromises the provisional matrix needed for wound repair. Adding platelet-like particles to hemophilia B plasma enhanced fibrin network density, clot strength, and stiffness, and improved fibroblast migration. This indicated that structural defects in hemophilic clots directly impair early wound healing and cell movement [50].

In vivo studies with hemophilia B mice consistently demonstrate delayed cutaneous wound healing compared with wild-type controls. Hemophilic mice exhibit slower wound closure, subcutaneous hematomas, recurrent bleeding, increased and persistent angiogenesis, and excess iron deposition—features consistent with ongoing microhemorrhage [51]. Single-dose FIX or activated FVII improves only select parameters, whereas daily factor replacement for several days is required to approximate normal healing [51,52]. Together, these findings show that impaired coagulation underlies delayed healing and that sustained hemostasis is essential for normal wound repair.

Hemophilia A models similarly show delayed wound healing with increased vascular permeability, abnormal neovascularization, and persistent inflammation. For instance, induced hemarthrosis in hemophilic mice demonstrated synovial vascular remodeling. FVIII correction only partially attenuated these abnormalities, and prolonged FVIII deficiency led to more severe vascular changes and impaired repair [53].

Clinical studies demonstrate that patients with hemophilia A and B have a higher risk of impaired wound healing, especially following surgery or trauma. Complications such as hematomas, infection, skin necrosis, and dehiscence are more likely with insufficient factor replacement [54]. A large retrospective study of patients with hemophilia A and B undergoing elective orthopedic procedures found a 6.5% incidence of early wound complications. Risk factors for impaired healing included the presence of factor inhibitors and surgery for pseudotumor. Higher factor consumption was noted in patients with infection, though not statistically significant [55].

#### 3.1.3. Leukocyte Adhesion Deficiency

##### Gene and Protein Function

Leukocyte adhesion deficiency (LAD) is a group of rare autosomal recessive disorders characterized by defects in cellular adhesion molecules that normally permit leukocytes to roll on blood vessel walls, undergo diapedesis between endothelial cells, and migrate to sites of infection [56]. The disease affects all leukocytes—from neutrophils to lymphocytes—impairing both innate and adaptive arms of the immune system [57,58].

There are 3 subcategories of LAD. In Type I LAD (LAD-I), mutations in the *ITGB2* gene encoding for the β2 subunit of CD18 lead to defective beta-2 integrin protein. In Type II LAD (LAD-II), there is a mutation to the *SLC35C1* gene, which encodes a GDP-fucose transporter. This reduces or eliminates Sialyl Lewis X expression on neutrophils, which are surface carbohydrate ligands for E- and P-selectin on activated endothelial cells. In Type III LAD (LAD-III), there is a mutation in the *FERMT3* gene, causing defective kindlin-3 and impairment of the integrin activation cascade [17].

##### Clinical Manifestations

LAD is rare, affecting 1 in 1 million people annually [56]. The most common clinical manifestations of LAD are recurrent bacterial infections and poor wound healing of the skin and mucosa. In all subcategories of LAD, there is absent or minimal purulence at the site of infection [59].

Specific features vary by subtype. In LAD-I, periodontitis and delayed separation of the umbilical cord can be seen [59]. LAD-II is associated with pulmonary symptoms such as pneumonia, bronchiectasis, and tuberculosis. Of note, infections in LAD-II are often less severe and frequent compared to those in LAD-I [56]. LAD-III can have symptoms of umbilical cord stump infection, bone fractures, and hematological abnormalities such as bleeding and bone marrow failure [17].

##### Wound Healing

Wound healing impairment in LAD is severe, persistent, and central to the clinical phenotype. Current literature shows that poor wound healing occurs in about 86% of patients with LAD [56]. Preclinical studies support possible mechanisms.

In vitro studies using leukocytes derived from patients with LAD confirm defective β2 integrin-mediated adhesion, chemotaxis, and transmigration. This resulted in impaired neutrophil recruitment and abnormal wound repair responses due to a lack of local inflammatory response, which is an essential step in wound healing [60,61].

In an in vivo study of CD18^−/−^ mice with full-thickness wounds, healing was markedly delayed during granulation and contraction because the absence of neutrophils at the wound site caused infiltrating macrophages to downregulate TGF-β signaling, blunting myofibroblast differentiation. Injection of TGF-β rescued this effect and led to wound closure [62,63]. Furthermore, transplantation of MSCs into CD18^−/−^ mice rescues the impaired wound healing phenotype. MSCs sense low TGF-β1 concentrations at wound sites and adaptively increase TGF-β1 release, a process regulated by TGF-β receptor signaling and microRNA-21/Smad7 pathways [64]. Thus, dysregulated TGF-β signaling may be a key contributor to the nonhealing wound microenvironment in patients with LAD.

Clinical case reports have also documented delayed wound healing in adult and pediatric patients with LAD [65,66]. For example, a multicenter study of 132 LAD patients found that those with lower CD18 expression (<2%) presented early in life with omphalitis, delayed umbilical cord separation, and recurrent skin and soft tissue infections, while those with higher CD18 expression (>30%) often presented later with chronic skin ulcers as the most common manifestation [67]. Additionally, a case report describes the resolution of a chronic cutaneous ulcer in a 30-month-old patient following successful hematopoietic stem cell transplantation [68].

#### 3.1.4. Sickle Cell Disease

##### Gene and Protein Function

Sickle cell disease (SCD) is an autosomal recessive blood disorder caused by a single A-to-T point mutation in the hemoglobin β-globin gene (*HBB*), creating a glutamate-to-valine substitution at the 6th amino acid of the resultant β-globin chain. In its wild-type state, two β-globin units assemble with two α-globin chains to form hemoglobin A (HbA). However, when two mutant β-globin chains pair with two normal α-globin chains, the resulting tetramer is hemoglobin S (HbS) [18,19].

The functional consequence of this single amino acid change is severe: the glutamate-to-valine substitution creates a hydrophobic patch on the protein’s surface, causing HbS to readily form long, stiff polymers with other HbS molecules inside of red blood cells (RBCs), particularly when hemoglobin is deoxygenated. As a result, RBCs containing HbS polymers will collapse into a rigid, sticky “sickle” shape, making them prone to hemolysis and creating a higher risk for small vessel occlusion and anemia [18,19].

##### Clinical Manifestations

SCD affects an estimated 7.74 million people globally [19,69]. Patients often present with a wide range of chronic symptoms such as daily chronic pain, pulmonary hypertension, renal dysfunction, avascular bone necrosis, and leg ulcers (LUs). Additionally, patients experience acute manifestations of SCD, which can include painful vaso-occlusive crises, acute episodes of hemolytic anemia, repeated infections (particularly from encapsulated organisms), cerebrovascular accidents, acute chest syndrome, acute kidney injury, and splenic injury [70].

##### Wound Healing

Impaired wound healing is strongly associated with SCD and most commonly manifests as chronic, nonhealing LUs. These LUs have a significant detrimental impact on quality of life and are present in up to 4% of SCD patients, though this incidence can vary widely across different regions and populations [19]. Although exact mechanisms underlying poor healing of LUs in SCD remain incompletely defined, evidence implicates multiple contributing pathways.

A mechanism that may contribute to dysfunctional wound healing is impaired angiogenesis. An in vivo mouse model of SCD showed that impaired blood and lymphatic angiogenesis in the wound bed, as well as low levels of CXCL12 (a chemokine for endothelial progenitor cell recruitment), may contribute to poor wound healing. The same study showed that endothelial progenitor cell (EPC) migration from the bone marrow to the wound site and proliferation were impaired; moreover, wound healing was rescued by injecting EPCs from healthy mice into the wounds. In contrast to the in vivo findings, EPCs isolated from SCD mice displayed normal intrinsic function in vitro. Complementary molecular analyses revealed reduced CXCL12 transcription in wound-derived keratinocytes and inflammatory cells, supporting a microenvironment-driven failure of EPC mobilization rather than an intrinsic defect in EPC function [71].

Further evidence from clinical studies and histological investigations provides more insight. A leading hypothesis is that intravascular hemolysis drives maladaptive vasoconstriction and downstream hypoperfusion. This is supported by clinical studies showing that hemolysis severity correlates with pulmonary hypertension, suggesting a hemolysis–vasculopathy link. Mechanistically, cell-free hemoglobin released during hemolysis scavenges nitric oxide (NO) and promotes reactive oxygen species (ROS) generation. This depletes NO, which is necessary for vasodilation, thereby reducing tissue perfusion needed for wound repair [72]. Subsequent vasoconstriction results in poor perfusion of downstream tissue and hypoxia that prevents a proper wound healing response [71,73].

Histological investigation of human skin biopsies taken from LUs in SCD patients shows microthrombi and fibrinous deposition in vessel walls in and surrounding the wound, causing luminal narrowing, venostasis, and microvascular occlusion [74].

Genetic factors have also been identified in cohort studies. SCD patients with human leukocyte antigen (HLA)-B35 and HLA-Cw4 were found to be 17 times more likely to develop LUs than SCD patients without these genotypes. Increased expression of certain microRNAs (miR-199a-5p and miR-144) and decreased expression of miR-126 are also associated with LUs. Finally, whole-exome sequencing identified an *FLG2* gene variant that was associated with an increased risk of developing LUs [75]. *FLG2* encodes filaggrin-2, a protein crucial for epidermal differentiation and skin barrier integrity. It is expressed in the outer layers of the epidermis, playing a key role in maintaining the skin’s barrier function and hydration, thus impacting wound healing [76].

#### 3.1.5. Thalassemia

##### Gene and Protein Function

Thalassemias are autosomal recessive disorders resulting from a quantitative defect in globin chain production. Beta-thalassemia is caused by over 200 mutations in the *HBB* gene, classified as beta(+) (reduced synthesis) or beta(0) (absent synthesis). This leads to decreased hemoglobin and microcytic anemia, with clinical severity classified by genotype, ranging from beta-thalassemia minor (heterozygous) to intermedia and major (homozygous or compound heterozygous) [20]. The resulting decreased hemoglobin synthesis causes ineffective erythropoiesis and intramedullary hemolysis. This triggers reactive bone marrow expansion, which leads to increased iron absorption and potentially iron overload, particularly in transfusion-dependent patients [20,77,78].

Alpha-thalassemia is caused by autosomal recessive mutations in the alpha-globin genes (*HBA1* and *HBA2*), affecting alpha-globin production. Disease severity depends on the number of the four alpha-globin genes inactivated. While deletion of one or two genes results in a silent carrier or alpha-thalassemia trait, respectively, the deletion of three genes causes Hemoglobin H (HbH) disease, characterized by abnormal beta-globin tetramers (β4). The deletion of all four genes is known as hemoglobin Bart’s hydrops fetalis syndrome (BHFS), defined by the formation of abnormal gamma-globin tetramers (γ4). Similarly to beta-thalassemia, the imbalance in globin chains results in hemolytic anemia, ineffective erythropoiesis, and bone marrow expansion [21,22,23].

##### Clinical Manifestations

About 68,000 children are born with beta-thalassemia major annually, and around 1.5% of the global population carries the mutation [79]. Patients with beta-thalassemia major often present in childhood with severe anemia, fatigue, growth delays, jaundice, and hepatosplenomegaly. Patients with beta-thalassemia minor may present later in life with jaundice, cholelithiasis, hepatosplenomegaly, bony deformities, leg ulcers, and pulmonary hypertension [20].

Globally, about 5–20% of the population carries an alpha-thalassemia mutation, and the prevalence of HbH disease is about 4–20 in 1000 births [21,22]. Alpha-thalassemia carriers with one or two mutant copies of the alpha-globin gene are often asymptomatic [80]. Patients with HbH disease have chronic hemolytic anemia that worsens under conditions of oxidative stress. BHFS manifests during late gestation, usually the 2nd or 3rd trimester, and is often lethal, causing fetal demise [81].

##### Wound Healing

Impaired wound healing, particularly the formation of chronic LUs, is associated with thalassemia and has been documented in numerous case reports and clinical series [82]. Although less researched than in sickle cell disease, the hypothesized underlying mechanism is similar: chronic hypoperfusion of the wound area driven by hemolytic anemia creates a hypoxic wound bed that is not conducive to healing [83]. Compounding this, iron overload further hinders granulation tissue formation by causing oxidative tissue damage, endothelial dysfunction, and vasculopathy [84].

Generally, preclinical in vitro or in vivo studies on wound healing in thalassemia are lacking, but some highlight oxidative stress, mitochondrial dysfunction, and immune abnormalities as potential pathways that may impair wound healing in thalassemia patients [85,86].

Clinical evidence suggests that the pathology is complex. A case series of beta-thalassemia major patients described hypercoagulability and thromboembolic complications, including recurrent arterial occlusions and venous thrombosis, that restrict tissue perfusion and contribute to poor wound healing [87].

Several clinical studies have reported experimental treatment modalities for thalassemic LUs. For instance, one prospective clinical study detailed the successful use of platelet-rich plasma gel in the treatment of 100 thalassemic LUs [88]. Additionally, in a case report of a patient with thalassemia intermedia, erythroexchange led to complete healing of a chronic, non-healing surgical wound refractory to standard care. This result supports the hypothesis that systemically correcting the underlying anemia and hemolysis can reverse impaired healing [84].

### 3.2. Premature Aging Disorders

Premature aging disorders are caused by genetic defects in genome stability mechanisms [89]. These syndromes are relevant to our discussion of genetic causes of wound healing because aging is associated with impaired wound healing due to multifactorial changes in cellular function, tissue architecture, and intercellular signaling. Data from animal models demonstrate that aged skin exhibits delayed wound closure, reduced keratinocyte and fibroblast proliferation, impaired angiogenesis, and dysregulated immune responses, including persistent inflammation and altered macrophage function [5,90]. These impairments can have serious implications on both the proliferative and remodeling phases of wound healing [91]. We highlight key progeroid syndromes—ataxia telangiectasia, Hutchinson-Gilford progeria syndrome and Werner syndrome—and summarize the preclinical and clinical evidence linking them to impaired wound healing.

#### 3.2.1. Ataxia Telangiectasia

##### Gene and Protein Function

Ataxia telangiectasia (A-T) is inherited in an autosomal recessive manner, affecting individuals with two pathogenic *ATM* alleles. Carriers typically remain asymptomatic. The wild-type ATM protein is a large nuclear and cytoplasmic serine/threonine kinase that coordinates the DNA damage response by phosphorylating hundreds of substrates involved in DNA repair, cell cycle checkpoints, apoptosis, and chromatin remodeling. ATM also regulates oxidative stress responses, mitochondrial function, RNA metabolism, and cellular homeostasis, with critical roles in neurons, immune cells, and other tissues. The ATM serine/threonine kinase also regulates mitochondrial autophagy, contributing to mitochondrial homeostasis [24].

Classic A-T is caused by loss-of-function mutations, while milder phenotypes are associated with missense and splicing site variations. Loss of ATM serine/threonine kinase activity disrupts DNA double-strand break signaling and cell-cycle checkpoints, leading to genomic instability with progressive neurodegeneration and immunodeficiency [24]. Additionally, reduced response to ionizing radiation and alkylating agents leads to malignant proliferation [92].

##### Clinical Manifestations

A-T is a rare neurodegenerative ataxia syndrome that affects the nervous and immune systems. It affects 1 in 100,000 to 1 in 40,000 people worldwide [24]. Classic A-T typically presents during the first decade of life with ataxia and ocular telangiectasias. Children may experience short stature and failure to grow. As patients age, they may develop skin and visceral telangiectasias, cutaneous atrophy, pigmentation changes, and hypertrichosis [92]. Cutaneous granulomas, often presenting as progressive, noninfectious lesions, may ulcerate and impair skin integrity, leading to chronic wounds [93]. Neurologically, tremors and mild to moderate cognitive impairment may develop. Immunodeficiencies affect about two-thirds of cases. Patients are at increased risk of malignancy, particularly lymphomas and leukemias [92]. Pulmonary disease occurs in more than 70% of patients [24].

##### Wound Healing

Preclinical data suggests that wound healing may be impaired due to defective DNA repair mechanisms and excessive inflammation secondary to ATM deficiency. Clinical evidence indicates that most wound complications arise from granuloma formation linked to immunodeficiency.

In vitro and animal studies support that ATM deficiency in A-T impairs wound healing through mechanisms involving defective DNA repair, increased oxidative stress, and dysregulated inflammatory responses. ATM-deficient fibroblasts show reduced proliferation and impaired DNA repair, suggesting a role for ATM in tissue regeneration; in a diabetic foot ulcer mouse model, restoring ATM expression by inhibiting miR-200 improved wound healing outcomes [94]. Additionally, ATM-deficient mouse models show that loss of ATM leads to prolonged inflammation, excessive neutrophil recruitment, and impaired tissue repair following injury. Treatment with reparixin, a CXCR1/CXCR2 antagonist, reduced neutrophil infiltration and tissue damage, suggesting that targeting inflammation may improve wound healing in A-T [95].

Clinical evidence regarding wound healing outcomes in patients with A-T is limited. However, there are case reports and clinical series documenting granuloma formation and granuloma-related wound complications. A case series of 8 patients with A-T reported 4 patients with cutaneous granulomas involving the face and trauma-prone areas of the upper and lower extremities [96]. One case study described a child with A-T whose non-infectious caseating granulomas and ulcerated plaques of the upper extremities were effectively treated with intravenous immunoglobulin therapy and topical mometasone. The authors also identified 22 other cases of A-T with cutaneous ulcerations in the literature [97]. Another study with a cohort of 44 A-T patients identified 8 cases with non-infectious cutaneous, bone, or synovial granulomas. Cutaneous granulomas did not respond to topical tacrolimus or corticosteroids. However, TNF inhibitors led to partial or complete regression in several patients, and hematopoietic stem cell transplantation (HSCT) achieved complete remission in one case [93]. Another case report also revealed resolution of destructive skin granulomas in a patient with AT after HSCT [98].

#### 3.2.2. Hutchinson-Gilford Progeria Syndrome

##### Gene and Protein Function

Classic Hutchinson-Gilford Progeria syndrome (HGPS) is caused by the mutation c.1824C>T (p.Gly608=) of *LMNA*, which activates a cryptic splice site in exon 11, producing progerin (lamin A Δ50), a prelamin A variant missing 50 amino acids near the C-terminus and therefore retaining farnesylation. It is inherited in an autosomal dominant pattern; however, almost all cases represent de novo mutations. Eleven nonclassical genotypes have been documented but are much rarer [99]. *LMNA* encodes A-type lamins, lamin A and lamin C, isoforms generated by alternative splicing, collectively referred to here as lamin A/C. Lamin A/C is a key structural component of the nuclear lamina, the inner lining of the nuclear membrane. By interacting with inner membrane proteins, transcription factors, and the genome, it plays a role in gene regulation and DNA replication and repair [25]. Progerin disrupts the nuclear lamina, causing nuclear blebbing, heterochromatin loss, and impaired chromatin organization. This results in genome instability, DNA damage, telomere dysfunction, and premature cellular senescence [100].

##### Clinical Manifestations

HGPS is a rare disorder that is characterized by premature aging. This condition affects about 1 in 20 million people worldwide [101]. Infants are typically born with a normal appearance but develop growth delays by the second year of life. Other clinical features typically develop during childhood and consist of distinct facial features [25]. Additionally, children may have short stature, sclerodermatous skin—tight and hardened—over the abdomen and upper thighs, progressive joint contractures, alopecia, subcutaneous lipodystrophy, low-frequency conductive hearing loss, and severe early-onset atherosclerosis [99].

##### Wound Healing

Clinical evidence for wound healing deficits in HGPS is sparse, yet preclinical work implicates multiple mechanisms that may compromise tissue repair.

In vitro studies of human cells demonstrate impairments in mechanisms essential for wound repair. Progerin expression in marrow-isolated adult multilineage inducible (MIAMI) cells was shown to disrupt stem cell self-renewal, proliferation, migration, and membrane elasticity, all critical for tissue repair [102]. Progeria-on-a-chip models using human induced pluripotent stem cells (iPSC)-derived smooth muscle cells from HGPS patients show exacerbated inflammatory responses and DNA damage under biomechanical strain, mimicking vascular aging and impaired repair [103].

Several in vivo studies with various HGPS murine models have suggested an association with HGPS and impaired wound healing. For instance, Zmpste24^−/−^ mice had significantly delayed wound closure as compared to age-matched male C57BL/6J wild-type mice. Additionally, Zmpste24^−/−^ mice demonstrated reduced cellular proliferation, increased DNA damage, impaired VEGF expression, decreased mobilization of bone marrow-derived vasculogenic progenitor cells, and reduced neovascularization, suggesting a link between progeroid mechanisms and impaired wound healing [104]. Lmna^G609G/G609G^ mice exhibited multiorgan fibrosis, inflammation, and dysfunction with evidence of reduced muscle regeneration and tissue remodeling, supporting the systemic impact of progerin on tissue repair [105]. In a tetracycline-inducible transgenic mouse model, forced expression of the LMNA c.1824C>T allele was associated with impaired epidermal wound healing and depleted epidermal stem cells, potentially explained by reduced levels of the stem-cell regulator p63 and evidence of premature senescence. Additionally, primary keratinocytes from the mice had reduced proliferative potential and ability to form colonies [106].

There are no clinical studies that have investigated or reported impaired wound healing in patients with HGPS, suggesting that the results of in vitro and in vivo studies do not translate clinically. Alternatively, the absence of clinical wound healing studies in HGPS is likely due to the rarity of the disease and the lack of reported wound healing complications. Such investigations to determine the clinical significance of animal models and in vitro studies are warranted.

#### 3.2.3. Werner Syndrome

##### Gene and Protein Function

Werner syndrome (WS) is caused by homozygous or compound heterozygous loss-of-function mutations in the *WRN* gene, with over 70 different pathogenic variants identified, inherited in an autosomal recessive pattern [26]. *WRN* encodes a RecQ DNA helicase, which uniquely possesses both 3′ to 5′ helicase and exonuclease activities. It contributes to genome stability by coordinating DNA double-strand break repair, enforcing replication checkpoint control, restarting stalled replication forks, supporting transcription, and maintaining telomeres [26]. *WRN* interacts with various DNA repair factors and is subject to regulatory phosphorylation in response to genotoxic stress [107].

Most pathogenic *WRN* mutations are nonsense or frameshift mutations that result in premature termination and production of truncated, nonfunctional protein. This mutant protein is often unstable, degraded rapidly, and fails to localize to the nucleus, which leads to loss of its DNA metabolic functions [108]. The lack of RecQ DNA helicase leads to accelerated replicative senescence and telomere attrition [109].

##### Clinical Manifestations

WS affects 1 in 100,000 individuals [108]. Unlike other premature aging disorders, affected individuals develop normally until adolescence, with an absent adolescent growth spurt being the first sign of disease, often recognized retrospectively [109]. By the second or third decade of life, WS patients experience skin atrophy, hair loss, and bilateral cataracts [26]. Patients may experience a pinched facial appearance, high-pitched voice, flat feet, thin limbs, and non-healing skin ulcers, typically around the elbows and ankles [26,107]. The condition increases the risk of type 2 diabetes mellitus, osteoporosis, hypogonadism, mesenchymal tumors, and atherosclerosis [26].

##### Wound Healing

Wound healing issues in WS are well-documented in the literature, as skin ulcers are a hallmark feature of the disease.

In vitro studies have shown that primary fibroblasts from WS patients exhibit accelerated cellular senescence, genomic instability, and impaired proliferative capacity, which are directly relevant to wound healing deficits [110,111]. Additionally, Tu et al. demonstrated that *WRN*^−/−^ MSCs showed downregulated hepatocyte growth factor (HGF), a key factor for cell proliferation and angiogenesis. Gene-corrected *WRN*^+/+^ iPSCs derived from WS patients differentiated into MSCs showed improved pro-angiogenesis and clonogenicity. Tu et al. also showed that when these cells were transplanted into a bone defect model in immunodeficient mice and a cutaneous wound model in diabetic mice, the gene-corrected *WRN*^+/+^ MSCs were associated with accelerated healing as compared to WS-MSCs [112]. This in vivo study is one of the few that directly assesses the relationship between WS and wound healing in an animal model.

Clinical evidence for wound healing issues has been largely documented in case reports. In some patients, a refractory skin ulcer is the initial presentation that, alongside other hallmark features, raised suspicion and led to diagnosis of WS [113,114,115]. A review of Japanese and English records of WS from 1996 to 2017 reported that about 40% of WS patients have skin ulcers. They are typically intractable and most often occur in the distal lower extremities [116]. In a cross-sectional analysis of 51 patients with WS enrolled in the Japanese Werner Syndrome Registry (2016–2022), 66.7% had skin ulcers. Older age and higher systolic blood pressure were independently associated with ulcer presence [117]. Such ulcers may also be associated with an underlying malignancy or foot calluses [116]. Histological analysis of skin surrounding ulcers in WS patients showed that skin ulcers are likely related to calcification within lymphatic vessels and ongoing lymphatic remodeling that impairs drainage [118].

Treatment consists of wound care, including debridement and negative pressure wound therapy (NPWT), and surgical approaches, such as the use of a dermal regeneration template, skin graft, flap, or amputation [116,119]. Other medical treatments have been investigated. The novel functional peptide SR-0379, known for its antimicrobial and wound-healing functions, was well tolerated and was shown to reduce leg ulcer size in 4 patients with WS [120]. Another novel functional peptide, AG30/5C, which has angiogenic and antimicrobial properties, was shown to improve a skin ulcer in a patient with WS that had been refractory to standard wound care [113]. A double-blind, randomized, crossover, placebo-controlled trial demonstrated that administration of the nicotinamide adenine dinucleotide (NAD+) precursor, nicotinamide riboside, was associated with a reduction in skin ulcer size among a variety of other end points related to wound healing, such as improvement in cardio-ankle vascular index and heel pad thinning and an improvement in renal function [121].

### 3.3. Connective Tissue Disorders

Connective tissue biology plays a crucial role in wound healing by providing both structural support and dynamic regulation of cellular activities throughout the repair process. The ECM—composed primarily of collagen, elastin, glycoproteins, and proteoglycans—serves as a scaffold for cell migration, proliferation, and differentiation, while also modulating the availability and activity of growth factors and cytokines essential for tissue regeneration and remodeling during the wound healing process [122].

In the following section, we examine how specific monogenic connective tissue disorders—cutis laxa, Ehlers-Danlos syndrome, epidermolysis bullosa, Loeys-Dietz syndrome, Marfan syndrome, osteogenesis imperfecta, and pseudoxanthoma elasticum—illuminate mechanisms of wound healing through their characteristic perturbations of ECM biology.

#### 3.3.1. Cutis Laxa

##### Gene and Protein Function

Cutis laxa is a group of disorders characterized by loose, inelastic skin. It is genetically heterogenous, resulting from pathogenic variants in multiple genes, including *ELN*, *FBLN5*, *FBLN4/EFEMP2*, *LTBP4*, and *ATP6V0A2*, that participate in elastic-fiber assembly, ECM secretion/processing, or intracellular trafficking [123]. The inheritance pattern is variable, ranging from autosomal dominant and autosomal recessive to X-linked; acquired forms also exist due to inflammatory or post-infectious elastolysis. The phenotypic severity and degree of systemic involvement are dependent on the specific genotype [27]. The genes associated with cutis laxa encode proteins that are either structural components of elastic fibers (e.g., elastin from *ELN*, fibulins from *FBLN5* and *FBLN4/EFEMP2*) or are required for their assembly, secretion, and crosslinking (e.g., elastic fiber maturation from *LTBP4* and ER-mediated ECM protein processing from *ATP6V0A2*) [27,124,125].

In the wild-type state, assembled elastic fibers are critical components of the ECM. Their primary function is to provide elastic recoil in tissues like the skin, lungs, blood vessels, and ligaments. They also organize the ECM and are involved in modulating signaling pathways, notably the TGF-β pathway [124,125].

Mutations in the genes associated with cutis laxa disrupt elastic-fiber biogenesis, leading to two primary consequences: a mechanical loss of tissue elasticity and disturbed matrix signaling. These issues collectively impair the normal process of tissue repair [124,125]. The downstream consequences are particularly relevant to wound healing and include the presence of fragmented or absent elastic fibers, which significantly reduces the skin’s recoil [126,127]. Furthermore, the structural integrity of the dermis is compromised, resulting in altered dermal thickness and the formation of smaller, disorganized collagen bundles [124,125]. On a molecular level, the disturbed matrix signaling increases dermal vascularity [125,126]. It also increases TGF-β activation, which drives excessive fibroblast activation, elevated protease expression, and subsequent aberrant remodeling of the matrix [27,124]. Finally, underlying secretory and trafficking defects directly impair the processing and secretion of essential ECM components, ultimately compromising proper matrix deposition and maturation [127].

##### Clinical Manifestations

Cutis laxa is a rare condition with a reported prevalence of approximately 1 in 4 million births [128]. Its hallmark clinical feature is loose, pendulous, and inelastic skin, which imparts a prematurely aged appearance. Beyond the skin, systemic manifestations can occur, including pulmonary emphysema, various forms of vascular involvement, and the development of hernias and diverticula. In recessive syndromic forms, variable neurodevelopmental features are present. Furthermore, acquired forms of cutis laxa present with localized findings [27].

##### Wound Healing

Wound healing problems are associated with cutis laxa due to the fundamental loss of elastic fiber integrity and deranged ECM organization. While specific epidemiological statistics on the prevalence of delayed or non-healing wounds in cutis laxa are not provided in the current literature, the underlying pathology, as described by preclinical studies, suggests potential impairment.

Cultured fibroblasts from patients with congenital cutis laxa show altered collagen expression and upregulation of MMPs, which are associated with degradation of elastic and collagen fibers in the skin. These findings suggest that increased MMP activity may impair wound healing by promoting excessive matrix breakdown [129]. Histologic reports from congenital and acquired cases show underdeveloped elastic fibers, reduced collagen bundle size, and increased dermal vascularity, all of which are features that correlate with softer supportive dermal tissue and reduced suture-holding capacity [130]. Consequently, when elastic fibers are fragmented or improperly assembled, wounds may close more slowly, demonstrate poor recoil under tension, and be prone to the recurrence of laxity even after primary surgical repair [126].

Murine models, especially fibulin-5 knockout mice, recapitulate features of cutis laxa, including marked defects in elastic fiber formation. Interestingly, studies indicate that the absence of elastic fibers does not significantly impair acute cutaneous wound healing in mice [131]. Long-term wound healing outcomes and effects on tissue integrity in animal models, particularly regarding scar quality, tensile strength, and chronic wound formation, have not shown major impairment [131,132].

Decreased tissue elasticity may be associated with postoperative complications. A case report of a patient undergoing surgical resection for acquired, localized cutis laxa reported no wound healing complications or recurrence after 6 months postoperatively [133]. However, another case report described the recurrence of skin laxity in a patient with congenital cutis laxa 10 years after a revision rhytidectomy [134]. Aside from case reports on complications following procedures to remove skin laxity, few reports about wound healing problems have been described.

#### 3.3.2. Ehlers-Danlos Syndrome

##### Gene and Protein Function

Ehlers-Danlos syndrome (EDS) is a heterogeneous group of genetic disorders comprising 13 subtypes and associated with 19 genes. This discussion focuses on the three most common forms, which account for 90% of cases: Classical EDS (cEDS), Vascular EDS (vEDS), and Hypermobile EDS (hEDS). Both cEDS and vEDS are inherited in an autosomal dominant pattern, caused by pathogenic variants in specific genes: cEDS is associated with variants in *COL5A1* and *COL5A2*, while vEDS is linked to variants in *COL3A1*. hEDS is inherited in an autosomal dominant pattern as well, but the molecular basis is currently unknown [28].

These genes encode various types of collagens, the major structural components of the ECM. Specifically, *COL5A1/COL5A2* encodes type V collagen, which is widely distributed in the dermis, tendons, and muscles and plays a critical role in type I collagen fibrillogenesis. The *COL3A1* gene encodes type III collagen, primarily found in the tunica media of blood vessels and hollow organs [28].

In their wild-type state, collagens are crucial for providing structural integrity across multiple connective tissues and organ systems; they are also integral to cell adhesion, chemotaxis, and migration and play a regulatory role in tissue remodeling during development and wound healing. In mutant forms, these gene variants lead to functionally defective collagens, resulting in structural defects and tissue fragility [28].

##### Clinical Manifestations

EDS affects an estimated 1 in 5000 to 1 in 100,000 people [135]. Generally, EDS presents with a combination of skin hyperelasticity, joint hypermobility, and blood vessel fragility [28].

The Classical subtype is characterized by marked skin involvement, including generalized joint hypermobility, skin hyperextensibility, abnormal wound healing, and atrophic scarring. Less common features include easy bruising, doughy skin, skin fragility, molluscoid pseudotumors, subcutaneous spheroids, abdominal wall hernias, and epicanthal folds [136]. The vascular subtype presents with thin, translucent skin that is less hyperelastic than cEDS skin. Blood vessels are fragile, resulting in extensive bruising, hematoma formation, and delayed wound healing after minor injury [28]. In hEDS, the defining features are impressive hyperextensibility of large and small joints, frequently leading to dislocation, swelling, arthritis, and chronic joint pain [137].

##### Wound Healing

Wound healing problems are strongly associated with EDS, particularly the cEDS and vEDS subtypes, though hEDS is not typically linked to such issues. While specific epidemiological statistics quantifying wound healing complications across all EDS patients are not readily available, extensive preclinical evidence and clinical documentation highlight significant impairment.

In vitro studies using cEDS fibroblasts reveal broad ECM disorganization—affecting type V and III collagens, fibronectin, and fibrillins—combined with a reduced migratory capacity and an aberrant wound healing phenotype. These cells also display an unusual survival mechanism involving α_*v*_ β_3_-integrin–epidermal growth factor receptor (EGFR) crosstalk that confers resistance to anoikis, enabling the cells to survive despite the loss of attachment to the ECM. Furthermore, murine models lacking *Col5a1* and *Col5a2* show a defective wound healing response and reduced cell migration [28].

In a comprehensive murine cEDS model, wounds demonstrate delayed closure, reduced and disorganized collagen fibrils, altered matrix mechanics, downregulated epidermal differentiation, and heightened inflammatory programs. Therapeutic strategies in this model, such as targeting the ECM–integrin axis or supplying normal fibroblasts, partially restored gene programs and accelerated closure, strongly implicating type V collagen deficiency and aberrant mechanosensing as key drivers of wound healing failure [138].

Several case reports have described wound healing issues in patients with EDS. A 41-year-old man with mild EDS sustained a right lateral foot wound. Despite drainage, debridement, elliptical excision, and reoperation, it was complicated by infection, partial skin necrosis, loosening nylon sutures, surgical wound dehiscence, and delayed healing. After initiating a collagen dressing, granulation improved and healing accelerated, with complete closure and no aberrant scarring by 4 months postoperatively [137]. A 33-year-old vEDS patient similarly suffered total abdominal wound dehiscence after laparotomy, achieving near-complete healing only after receiving intravenous allogeneic mesenchymal stromal cells (MSCs) and high-dose Vitamin C [139].

Supporting these individual accounts, larger cohort studies confirm that fragile tissue repair is a central feature. A cross-sectional study of 75 molecularly confirmed cEDS patients found that abnormal/atrophic scarring was the most frequent and characteristic cutaneous sign, highlighting disordered scarring as a key manifestation [140]. Similarly, a large retrospective cohort from the UK National Diagnostic Service analyzed 180 patients with molecularly confirmed vEDS and reported that tissue fragility and poor wound healing are major clinical challenges, especially when complicated by invasive or emergency surgical procedures [141].

#### 3.3.3. Epidermolysis Bullosa

##### Gene and Protein Function

Epidermolysis bullosa (EB) is a collective term for rare inherited skin disorders defined by tissue fragility and blistering, categorized into four main subtypes based on the level of dermal-epidermal junction separation: Epidermolysis Bullosa Simplex (EBS), Junctional EB (JEB), Dystrophic EB (DEB), and Kindler EB (KEB) [29,30].

The specific genes and inheritance patterns vary by subtype: EBS, the most common, makes up 70% of cases. It is autosomal dominant or recessive and caused by variants in *KRT5*, *KRT14*, *PLEC*, *DST*, *CD151*, *EXPH5*, and *COL7A1* [30].

*KRT5* and *KRT14* encode keratin 5 and keratin 14, respectively, which are intermediate filaments expressed in basal epidermal cells, forming a heterodimer and providing structural support to maintain the proliferative basal keratinocyte layer of stratified epithelia [31]. *PLEC* encodes plectin and *DST* encodes dystonin, both of which connect intermediate filaments to hemidesmosomes [30]. *CD151* encodes a cell surface glycoprotein that forms complexes with integrins and other transmembrane 4 superfamily proteins. Its expression is localized to hemidesmosomes and is necessary for cell adhesion. *EXPH5* encodes exophilin-5, a RAB27b GTPase effector protein, which participates in cell membrane trafficking and vesicle formation [32].

DES is caused by fragility below the lamina densa due to mutant *COL7A1*, which encodes type VII collagen, a major component of anchoring fibrils that support dermal-epidermal adhesion [33].

JEB is caused by mutations to *LAMA3*, *LAMB3*, or *LAMC2*, which result in defective laminin 332. Laminin 332 is a key component of anchoring filaments of the basement membrane and is used in keratinocyte migration during wound healing. JEB may also be caused by mutations to *ITGA6*, *ITGB4*, or *ITGA3*, which encode integrins that are necessary for ECM adhesion, hemidesmosome integrity, keratinocyte function, and cell signaling. Lastly, *COL7A1* may be mutated as well [30].

KEB is associated with a mutation to *FERMT1*, which encodes kindlin-1 protein, essential for keratinocyte stability and integrin activation [30].

##### Clinical Manifestations

EB affects approximately 10 in 1 million people worldwide and typically presents at birth or in early childhood [30]. The severity and specific manifestations differ across subtypes. EBS is characterized by non-scarring blisters and erosions in response to minor trauma, often limited to the hands and feet, but can be widespread. Other features include hyperkeratosis of palms/soles and nail dystrophy [32].

DEB involves blistering and erosion that heals with milia and scarring, frequently leading to severe complications like contractures, pseudosyndactyly, and esophageal strictures. Patients with severe recessive DEB (RDEB) face a lifetime risk of squamous cell carcinoma (SCC) greater than 90% [33]. JEB typically features non-scarring blisters across the body and mucous membranes, with severe forms often involving significant granulation tissue formation [142]. KEB presents with blistering, diffuse cutaneous atrophy, photosensitivity, and poikiloderma. Patients are also at risk for SCC [143].

##### Wound Healing

Wound healing problems are strongly and universally associated with EB and are considered a prominent, defining feature of the disorder. Clinically, about 32% of patients across all subtypes have wounds affecting more than 30% of their body surface area, requiring constant, resource-intensive wound care [144].

Preclinical evidence supports this impairment, as in vitro studies show that keratinocytes and fibroblasts, particularly from RDEB patients, exhibit impaired migration, abnormal ECM deposition, and dysregulated inflammatory signaling [13]. Wounds also demonstrate reduced tensile strength, as shown in a 3D in vitro model of the dermoepidermal junction in which keratinocytes and fibroblasts derived from RDEB patients exhibited significantly decreased mechanical adhesion compared with controls [145]. Furthermore, in vivo RDEB models (type VII collagen-hypomorphic mice) demonstrate delayed closure in full-thickness wounds, characterized by prolonged inflammation and deficient granulation tissue [146].

Clinical evidence for impaired healing includes chronic non-healing wounds, recurrent blistering and erosions, secondary infections, excessive inflammation, hypertrophic granulation tissue, scarring, and contractures [147]. These wounds are highly susceptible to secondary infection, most frequently *Staphylococcus aureus* with *Staphylococcus epidermidis* and *Pseudomonas aeruginosa*, which contributes to sepsis—a leading cause of mortality. Chronic wounds also contribute to chronic anemia, hypoproteinemia, delayed growth, and increased risk of aggressive SCC, all of which further contribute to delayed healing [148].

Recent randomized controlled trials (RCTs) have evaluated new therapies for wound healing in EB. For example, the phase 3 VIITAL trial demonstrated that prademagene zamikeracel, a topical gene therapy, improved healing of large, chronic RDEB wounds, which are otherwise refractory to standard care [149]. Another phase 3 trial showed that Oleogel-S10 (birch triterpenes) accelerated wound closure in EB compared to control gel [150]. Additionally, a pilot study showed that the use of a hypochlorous acid-based solution on chronic wounds in EB patients showed a significant reduction in *S. aureus* colonization, alongside increased microbial diversity and reduction in wound size [148].

#### 3.3.4. Loeys-Dietz Syndrome

##### Gene and Protein Function

Loeys–Dietz syndrome (LDS) arises from pathogenic variants in *TGFBR1*, *TGFBR2*, *TGFB2*, *TGFB3*, *SMAD2*, or *SMAD3* (with rare autosomal recessive forms due to *IPO8*); most LDS is autosomal dominant with variable expressivity and high vascular penetrance [34,151]. These genes encode TGF-β pathway ligands (*TGFB2/3*), receptors (*TGFBR1/2*), and intracellular mediators (*SMAD2/3*) [34]. In the wild-type, canonical TGF-β signaling regulates cell proliferation and differentiation, ECM production, inflammation, and tissue remodeling [34]. Pathogenic variants perturb TGF-β pathway dynamics—often with paradoxical hyperactivation—disrupting ECM homeostasis, elastic-fiber architecture, and vascular integrity [34]. Affected connective tissues demonstrate elastic-fiber fragmentation, abnormal collagen–elastin ratios, and increased proteolysis [152]. Because TGF-β directs inflammation, fibroblast activation, and ECM deposition during repair, these signaling abnormalities alter fibroblast and myofibroblast responses after injury [34].

##### Clinical Manifestations

LDS is rare with an unknown prevalence [153]. Patients present with aggressive, diffuse aortopathy involving arterial tortuosity, aneurysm formation, and dissection. Characteristic craniofacial findings include hypertelorism and a bifid uvula or cleft palate. Skeletal manifestations overlap with those of Marfan syndrome (see Section 3.1.5) and include scoliosis, pectus deformities, and joint hypermobility. Cutaneous features such as translucent skin, easy bruising, and dystrophic or atrophic scarring further reflect underlying connective tissue fragility [153,154].

##### Wound Healing

Abnormal wound healing—including dystrophic scarring and delayed repair—is a characteristic and reported feature of LDS; however, large clinical studies are lacking.

In vitro fibroblast studies showed that cells with dominant negative SMAD3 variants demonstrated reduced extracellular matrix formation compared to control cell lines [155]. Additionally, fibroblasts with *TGFBR1* mutations showed decreased expression of elastin and fibulin 1 genes with impaired deposition of elastic fibers, while those with *TGFBR2* mutations exhibited intracellular accumulation of collagen type I with otherwise normal elastic fiber production [156].

A patellar tendon transection model in LDS mice showed delayed healing and weaker tissue repair. Baseline morphologic tendon differences may have predisposed mice to tendon/ligament dysfunction and a blunted reparative response after injury as compared to controls [157]. LDS mouse models also demonstrated that angiotensin II–dependent augmentation of TGF-β signaling drives postnatal disease progression, supporting a paradigm in which excess TGF-β activity impairs matrix homeostasis and tissue integrity, a mechanism plausibly extending to fragile, failure-prone wound repair in LDS [158].

LDS patients—especially after cardiovascular procedures—exhibit higher rates of postoperative complications, including bleeding, delayed healing, wound dehiscence, and anastomotic failure [159]. Case reports and institutional series describe non-healing median sternotomy wounds and other surgical-site complications necessitating advanced wound care. For instance, a patient with LDS experienced 10 months of delayed wound healing following a median sternotomy for valve-sparing aortic root surgery. The patient ultimately healed with incisional negative pressure wound therapy (NPWT) [160].

#### 3.3.5. Marfan Syndrome

##### Gene and Protein Function

Marfan syndrome (MFS) results from pathogenic *FBN1* variants and is typically inherited in an autosomal dominant pattern with variable expressivity and pleiotropy [35,161]. *FBN1* encodes fibrillin-1, which assembles into microfibrils that support tissue mechanics and scaffold elastin while regulating TGF-β bioavailability via latent TGF-β–binding proteins [35,36]. Pathogenic variants in MFS impair microfibrillogenesis and TGF-β sequestration, elevating local TGF-β signaling and altering fibroblast behavior, ECM remodeling, and apoptosis [36,161]. The weakened ECM architecture decreases tensile strength and alters the mechanical cues sensed by keratinocytes, fibroblasts, and myofibroblasts—cells essential for wound closure and matrix deposition [162].

##### Clinical Manifestations

MFS affects about 1–2 in 10,000 people [163]. Clinically, it presents withmultisystem involvement. The cardiovascular system is most critically affected, with progressive aortic root dilation that predisposes to aneurysm and aortic dissection [164]. Skeletal features include tall stature, arachnodactyly, scoliosis, and chest wall deformities such as pectus excavatum or carinatum [165]. Ocular findings commonly include ectopia lentis, lens subluxation, and severe myopia, with an elevated risk of retinal detachment [166]. The integumentary system is also affected, as patients often display striae and thin, fragile skin [162].

##### Wound Healing

Although MFS features connective-tissue fragility, it is not typically linked to overt impairments in wound healing [167].

However, preclinical evidence may support an association between MFS and impaired wound healing. A scratch wound assay demonstrated impaired cell migration in Marfan fibroblasts, indicating altered directional migration necessary for wound closure. The same study demonstrated significantly increased apoptosis in Marfan fibroblasts [168]. Additionally, in a ligature-induced periodontal injury model, *Fbn1*^C1039G/+^ (Marfan) mice demonstrated comparable initial tissue destruction to wild type but significantly delayed soft-tissue and bone regeneration with persistent inflammatory infiltrates and sustained MMP-9 and TNF-α expression, indicating that fibrillin-1 insufficiency impairs connective-tissue wound healing [169].

A 2022 case report by Recker and colleagues described a patient with an atypical form of MFS who experienced wound dehiscence following cranial vault reconstruction for craniosynostosis. Histologic analysis revealed disorganized collagen fibers and an aberrant fibroblast response, consistent with the known consequences of fibrillin-1 deficiency and elevated TGF-β signaling. The authors concluded that excessive TGF-β activity and poor ECM organization disrupted normal healing stages, emphasizing the need for meticulous surgical planning, gentle tissue handling, and extended postoperative monitoring in patients with MFS [170]. Larger clinical studies documenting wound healing impairment in MFS are not available.

#### 3.3.6. Osteogenesis Imperfecta

##### Gene and Protein Function

Osteogenesis imperfecta (OI) most commonly results from heterozygous dominant *COL1A1* or *COL1A2* variants (~85% of classic cases), with additional dominant or recessive defects in collagen-processing genes producing a spectrum from mild (Type I) to perinatal lethal (Type II) disease [171]. *COL1A1/COL1A2* encode the α1(I)/α2(I) chains of type I collagen, the principal fibrillar collagen in bone, skin, tendon, dentin, and other connective tissues. In the wild-type, precise synthesis, post-translational modification, and extracellular assembly of the heterotrimer ensure matrix tensile strength and proper mineralization. Pathogenic variants cause quantitative deficiency or qualitative structural defects, compromising fibrillogenesis, weakening bone and soft-tissue matrices, and altering fibroblast–matrix signaling, MMP activity, and collagen deposition/remodeling—processes essential for effective wound repair [37]. Given type I collagen’s central role in dermal ECM organization, its deficiency or dysfunction disrupts all phases of repair. It lowers tensile strength, alters fibroblast migration and integrin-based signaling, and impairs crosslinking. The result is wound dehiscence, delayed healing, and a higher risk of mechanical failure at closure sites [37]. Notably, specific genotypes, such as *IFITM5* (Type V), can yield aberrant repair phenotypes, underscoring that regeneration may be pathologically exaggerated or insufficient depending on the molecular defect [172].

##### Clinical Manifestations

OI has a prevalence of about 0.3–0.7 per 10,000 births [171]. It primarily affects the skeletal system, with hallmark features including recurrent low-trauma fractures, long-bone deformities, vertebral compression fractures, and short stature in moderate to severe forms [173]. Dentoalveolar involvement commonly presents as dentinogenesis imperfecta, with opalescent, structurally compromised dentin that predisposes teeth to rapid wear [171]. Progressive hearing loss is common [174]. Some individuals exhibit mild skin laxity or fragility, reflecting the role of type I collagen as a dermal scaffold. These integumentary abnormalities can reduce wound tensile strength and alter scar architecture, predisposing to delayed healing [37].

Those with OI Type V frequently develop hypertrophic or hyperplastic callus formation after fractures or surgical procedures. These exuberant repair lesions can mimic infection or neoplasia and are often accompanied by inflammatory changes [175].

##### Wound Healing

Across preclinical and clinical studies, wound healing impairment in OI most often manifests as delayed or abnormal bone fracture healing.

In vitro studies are limited, with studies focusing on collagen production rather than wound healing processes. However, in vivo models provide more insight. For instance, both dominant and recessive OI mouse models (*Col1a2* and *Crtap*) show delayed fracture healing, with reduced callus size, altered cartilage distribution, and impaired biomechanical strength of the healed bone. These models also demonstrate abnormal chondrocyte maturation and upregulated TGF-β signaling during healing, which may contribute to the delayed repair process [176]. Studies modeling therapies in OI mice show that anabolic and antiresorptive agents (e.g., BMP-2, bisphosphonates) can modulate fracture healing, but BMP-2 alone is less effective in OI settings [177].

Clinical studies emphasize that connective tissue fragility in OI complicates wound management. In a decade-long surgical review, Georgescu and colleagues observed that patients with OI often experience delayed wound closure, wound dehiscence, and hematoma formation due to the inability of dermal and subcutaneous tissues to retain sutures under tension. Successful healing required atraumatic tissue handling, tension-free closure, and extended postoperative immobilization [178]. Interestingly, studies in pediatric patients receiving intravenous bisphosphonates for moderate to severe OI revealed altered callus dynamics and delayed osteotomy healing, underscoring the interplay between pharmacologic modulation of bone turnover and intrinsic collagen defects [179].

#### 3.3.7. Pseudoxanthoma Elasticum

##### Gene and Protein Function

Pseudoxanthoma elasticum (PXE) is driven primarily by biallelic *ABCC6* variants, with overlapping ectopic-mineralization phenotypes from *ENPP1* and *GGCX*. Inheritance is classically autosomal recessive with variable expressivity, and rare autosomal dominant and digenic cases are reported, likely influenced by modifiers [180,181]. *ABCC6* encodes an ATP-binding cassette transporter expressed mainly in the liver and kidney. In the wild type, *ABCC6* activity sustains circulating anti-mineralization capacity—by exporting substrates and modulating inhibitors of calcification—thereby preventing elastic-fiber calcification. Loss of function reduces systemic anti-mineralization factors, leading to dystrophic calcification of elastic fibers in skin, eyes, and vasculature [38,39]. Histopathology shows mid-dermal and arterial-media elastic-fiber calcification with elastorrhexis, causing fragmentation, stiffening, and loss of recoil, which diminishes suture retention and predisposes to cracking or extrusion of calcified fragments [182,183]. Vascular calcification and peripheral arterial disease further impair perfusion and oxygenation, compounding defects in wound repair [184].

##### Clinical Manifestations

PXE is estimated to affect roughly 1–4 in 100,000 individuals, with a slight female predominance [180]. PXE presents with yellowish papular skin lesions and lax, redundant folds. Ophthalmologic findings include *peau d’orange* and angioid streaks with risk of choroidal neovascularization. Clinical severity and onset are variable [180,185]. PXE alters both the mechanical and biochemical properties of the dermis. Mineralized elastic fibers become brittle, decreasing skin elasticity and compromising tensile strength and suture holding. Patients may also develop systemic vascular disease, which impairs perfusion, reducing oxygen and nutrient delivery to healing wounds [180].

##### Wound Healing

Preclinical In vitro studies consistently demonstrate that PXE is associated with abnormal fibroblast function, ECM remodeling, oxidative stress, and mineralization, all of which are likely to contribute to impaired wound healing in this disorder. However, in vivo studies directly studying wound healing in PXE are limited. Clinically, PXE patients demonstrate a variable but notable risk of delayed wound closure and surgical repair challenges [180].

In in vitro models, PXE fibroblasts show exaggerated migration but reduced myofibroblast contractility and impaired differentiation compared to controls, with diminished induction of myofibroblast markers (α-smooth muscle actin, xylosyltransferase-I) and poor TGF-β responsiveness. These abnormalities suggest a pathological deviation in wound healing processes and ECM remodeling in PXE skin [186]. PXE fibroblasts also have increased MMP expression and a pro-calcification phenotype, further implicating dysregulated ECM turnover in impaired repair [187].

Cutaneous correction procedures generally yield satisfactory outcomes, though isolated cases document extrusion of calcified particles, local hardening at suture sites, and delayed healing in heavily mineralized areas [183,188]. A case series of 9 patients undergoing cosmetic surgery for severe cutaneous stigmata of PXE described variable healing outcomes, including tissue friability, delayed repair, and occasional keloid formation [189]. These findings reflect the underlying histopathology and demonstrate real-world implications of dermal mineralization for wound integrity. Ophthalmic surgeries in PXE carry higher rates of intraoperative and postoperative complications, emphasizing the importance of specialized preoperative evaluation [190].

## 4. Polygenic Diseases

While the preceding sections have detailed the profound impact of monogenic disorders on tissue repair, these conditions represent a relatively small fraction of chronic wound etiologies. A far more significant burden stems from polygenic and multifactorial diseases, which are the dominant drivers of impaired wound healing in the general population. Polygenic diseases result from the complex interplay between modest-effect variations in numerous genes and significant contributions from environmental and lifestyle factors [191]. In practice, this complex interplay is quantified from GWASs, which identify variant loci contributing to disease pathogenesis, and polygenic risk scores (PRS), which aggregate these variant loci into a single inherited-risk estimate.

The polygenic diseases discussed in this section—though distinct in their primary clinical presentation—often converge on a few shared, core pathophysiological pathways that are detrimental to wound healing. These conditions frequently co-exist, creating a synergistic network of pathology that includes chronic systemic inflammation, endothelial dysfunction, macro- and micro-vascular ischemia, oxidative stress, and disordered ECM remodeling [192]. This section and Table 2 discuss key, highly prevalent chronic diseases that are known risk factors for impaired wound healing—chronic kidney disease, diabetes mellitus, hypertension, obesity, and peripheral artery disease—and highlight genetic loci potentially implicated in such pathology.

### 4.1. Chronic Kidney Disease

#### 4.1.1. Gene and Protein Function

Chronic kidney disease (CKD) is associated with multiple polygenic traits that affect glomerular integrity, tubular ion handling, and renal development. The highly polygenic architecture of kidney function is illustrated by a recent multi-ancestry GWAS of kidney function in approximately 2.2 million individuals that reported 1026 independently associated loci and used multi-omic kidney data to prioritize coding and regulatory mechanisms [208].

The genetic variants associated with CKD do not directly impair wound healing. Instead, these upstream genetic pathways shape the disease state, which, via uremia, anemia, vascular dysfunction, and inflammation, ultimately interferes with tissue repair.

Several gene families contribute to CKD susceptibility. Among glomerular and podocyte-associated genes, *APOL1* risk variants are strongly linked to CKD in individuals of African ancestry. *APOL1* variants produce toxic gain-of-function ion channels in podocytes, which further lead to cellular injury [209]. In a multi-ancestry genome-wide PRS analysis that combined common-variant associations for kidney function with APOL1 risk genotypes, each standard deviation increase in the PRS was associated with an odds ratio of 1.46 in European-ancestry cohorts and 1.32 in African-ancestry cohorts. Individuals in the top 2% of the score distribution had roughly threefold higher odds of CKD compared with the rest of the population [210].

Multiple genes regulating renal ion handling are also associated with CKD. The *UMOD* gene encodes uromodulin, a protein that modulates NKCC2 and ROMK transporter activity. Elevated expression of uromodulin enhances sodium reabsorption, which predisposes individuals to salt-sensitive hypertension and CKD [193].

Two other mutations, *PKD1* and *PKD2*, are associated with CKD and cystic kidney disease, respectively, and map to polycystin 1 and polycystin 2, respectively. Together, these proteins regulate tubular mechanosensation and calcium signaling. Their loss-of-function variants trigger cystogenesis and progressive renal failure, characteristic of autosomal-dominant polycystic kidney disease [194].

Genes that maintain the glomerular basement membrane also contribute to CKD risk. The glomerular basement membrane is primarily composed of type IV collagen alpha chains encoded by *COL4A3*, *COL4A4*, and *COL4A5*. Mutations in these genes destabilize the glomerular basement membrane (GBM) scaffold and underlie Alport syndrome, which leads to proteinuria, hematuria, and progressive chronic disease [195].

Finally, mutations in the *HNF1B* gene are implicated in CKD. HNF1B is a critical transcription factor for nephrogenesis, with haploinsufficiency leading to renal cysts, canal formations, and early-onset diabetes [196]. Together, these variants in Table 2 influence wound outcomes indirectly by increasing the likelihood of developing CKD and its systemic sequelae rather than altering tissue repair pathways in isolation.

#### 4.1.2. Clinical Manifestations

CKD is a major global health problem, affecting an estimated 788 million adults worldwide in 2023 [211]. It presents a progressive decline in glomerular filtration rate and is marked by metabolic, vascular, and inflammatory derangements that collectively impair tissue repair [212]. Clinically, CKD is characterized by uremia, anemia of chronic disease, mineral bone disorder, pruritus, and edema. These abnormalities contribute to tissue hypoxia, oxidative stress, and reduced immune competence. CKD also alters skin physiology, as patients exhibit atrophy of dermal connective tissue, all of which predisposes them to barrier dysfunction and impaired wound closure [213].

#### 4.1.3. Wound Healing

CKD is a well-known risk factor for impaired wound healing and complications such as infection and the need for amputation. Importantly, the genetic variants themselves do not directly impact wound repair. Rather, in vitro and in vivo studies have demonstrated that the systemic consequences of CKD interfere with the healing process and create a chronic state of inflammation, oxidative stress, vascular dysfunction, and impaired immunity. Correspondingly, GWAS and PRS studies directly linking inherited CKD risk to wound healing outcomes have not been reported.

Multiple CKD-specific barriers slow repair. Human endothelial cells, when exposed to uremic toxins (indoxyl sulfate and p-cresyl sulfate), suppress endothelial proliferation, impair endothelial-progenitor function and neovascularization, and increase ROS. Collectively, these processes stall wounds in the inflammatory phase [214]. Anemia-driven hypoxia further reduces oxygen delivery for fibroblast and keratinocyte metabolism and collagen hydroxylation. Murine models have found that chronic inflammation due to CKD leads to an upregulation of inflammatory cytokines, which suppresses erythropoiesis and leads to poor oxygen delivery [215].

The severity of CKD impacts wound healing complications. A retrospective study reported an increased hazard ratio for foot ulceration in patients with CKD stage 4–5 and dialysis-dependent patients as compared to patients with CKD stage 3. They observed similar increases in amputation risk [216]. Additionally, as many patients with CKD also have other comorbidities like diabetes, hypertension, and peripheral artery disease, wound healing is further impaired, and infection risk in non-healing ulcers is increased [217]. These pathogenic mechanisms coalesce and drive clinical outcomes. For instance, a large United States database study found that CKD patients had increased surgical site infection risk and wound healing complications after major orthopedic surgery [218].

### 4.2. Diabetes Mellitus

#### 4.2.1. Gene and Protein Function

The genetic variants associated with diabetes mellitus do not directly disrupt wound healing. Instead, they influence key pathways involved in immune tolerance, β-cell function, insulin production, and metabolic regulation. These genetic effects shape susceptibility to type 1 diabetes mellitus or type 2 diabetes mellitus, and it is the resulting metabolic and vascular abnormalities that impair tissue repair.

Type 1 diabetes mellitus (T1DM) is driven by autoimmune destruction of pancreatic β cells, and genetic susceptibility involves genes related to MHC class II, *PTPN22*, and *CTLA4*. The strongest determinants are MHC class II alleles—particularly *DR3*, *DR4*, *DQ2*, and *DQ8*—which encode antigen-presenting molecules on immune cells. These variants enhance the presentation of self-peptides from pancreatic β-islet cells to CD4^+^ T-cells, promoting autoreactivity. Variants in two more immune genes also drive susceptibility to T1DM. *PTPN22* encodes the lymphoid-specific tyrosine phosphatase (Lyp), a negative regulator of T-cell receptor signaling. Risk variants such as R620W attenuate this inhibitory “brake,” permitting escape of autoreactive T-cells from tolerance checkpoints. The second gene, *CTLA4*, functions as an immune checkpoint that restrains T-cell activation, and susceptibility variants reduce inhibitory signaling, further amplifying autoreactive T-cell activity [197]. Together, these variants raise the risk of autoimmune β-cell loss. The resulting absolute insulin deficiency is the primary driver of downstream changes, including chronic hyperglycemia, immune dysfunction, and microvascular damage, which interfere with wound healing.

Type 2 diabetes mellitus (T2DM) results from a combination of insulin resistance and eventual β cell failure, and the genetic variants involved regulate insulin, adipocyte function, and energy balance. Large-scale GWAS underscore that T2DM risk is broadly polygenic rather than attributable to a small set of loci. One analysis reported 1289 independent association signals mapped to 611 loci, with signals clustering into mechanistic groups enriched in pancreatic islets, adipocytes, endothelial cells, and enteroendocrine cells [219]. Complementing locus discovery, polygenic scores provide a quantitative measure of inherited genetic susceptibility. In a multi-population evaluation of a T2DM PRS, each standard deviation increase in the score was associated with a 1.78-fold higher odds of T2DM across populations, and individuals in the top decile had 2.37-fold higher odds compared with those in the middle of the distribution. The PRS also improved discrimination beyond age, sex, body mass index (BMI), and ancestry principal components [220].

Genetic risk loci associated with T2DM polygenic susceptibility include *TCF7L2*, *SLC20A8*, *KCNJ11*, *PPARG*, and *FTO*. *TCF7L2* encodes a transcription factor in Wnt signaling, which regulates proglucagon expression in intestinal L-cells, affecting glucagon-like peptide-1 (GLP-1) production and insulin secretion [198]. *SLC30A8* encodes ZnT8, a zinc transporter in pancreatic β-cell granules. This provides zinc for insulin crystallization and storage. Loss-of-function variants surprisingly protect against T2DM, suggesting ZnT8 activity modulates β-cell stress and insulin release efficiency [202]. *KCNJ11* encodes Kir6.2, a subunit of the ATP-sensitive K^+^ channel in β-cells. Mutations affect the insulin release threshold. Variants raise the threshold for insulin release, impairing insulin secretion [199]. *PPARG* encodes peroxisome proliferator–activated receptor gamma (PPAR-γ), a nuclear receptor regulating adipocyte differentiation and lipid metabolism, which controls insulin sensitivity in adipose tissue [200]. *FTO* encodes an N^6^-methyladenosine demethylase that is associated with regulation of energy homeostasis and appetite control. Risk alleles are linked to obesity via altered hypothalamic control of food intake [201]. Together, these genes shape the metabolic environment that leads to dyslipidemia, hyperglycemia, and chronic inflammation.

#### 4.2.2. Clinical Manifestations

Diabetes mellitus (DM) is a highly prevalent chronic disease, affecting an estimated 561 million people worldwide in 2023 [221]. T1DM makes up 5–10% of cases [222].

T1DM typically presents in childhood or adolescence with abrupt polyuria, polydipsia, and weight loss. Over time, microvascular complications, such as retinopathy and nephropathy, arise. Patients are also at an increased risk for skin and soft-tissue infections and fungal infections. The primary issues are poor cellular energetics and immune dysfunction, leading to delayed granulation and epithelialization. Peripheral neuropathy may emerge, leading to plantar trauma and chronic ulcers [222].

The onset of T2DM is typically in adulthood and often presents in those with metabolic syndrome features, such as obesity, hypertension, and dyslipidemia. Chronic hyperglycemia and hyperinsulinemia lead to chronic endothelial dysfunction and inflammation. Vascular complications include retinopathy, neuropathy, and nephropathy. Patients may also experience chronic ulcers that are further complicated by concomitant kidney disease and vascular dysfunction [221].

#### 4.2.3. Wound Healing

Impaired wound healing in diabetes is well documented in the preclinical and clinical literature. Wound impairment arises from the metabolic and vascular consequences of chronic hyperglycemia, and the pathogenesis of wound healing in diabetes is multifactorial.

Hyperglycemia destabilizes hypoxia-inducible factor 1-α (HIF-1α), which reduces VEGF signaling and impairs angiogenesis, a finding demonstrated in in vivo mouse models [223]. At the same time, hyperglycemia also promotes the formation of advanced glycation end products (AGEs), which stiffen collagen, disrupt extracellular matrix remodeling, and activate RAGE signaling pathways that perpetuate NF-κB–driven inflammation [224]. These changes prevent wounds from transitioning out of the inflammatory phase and contribute to chronic non-resolving wounds. Additionally, hyperglycemia increases reactive oxygen species, which damage keratinocytes and fibroblasts, slowing proliferation and migration needed for re-epithelialization. Immune function is also compromised: an in vivo mouse study showed that neutrophils have defective chemotaxis and phagocytosis [225], while a review found that excessive neutrophil activity and NLRP3 inflammasome activation keep wounds in a pro-inflammatory state rather than transitioning to resolution [226].

Additionally, sensory loss due to neuropathy predisposes to repeated unnoticed trauma and micro- and macrovascular disease, particularly in T2DM. This limits perfusion and oxygen delivery. Clinically, these defects stall the normal healing phases, resulting in chronic, infection-prone wounds such as diabetic foot ulcers (DFUs). In those with DM, the lifetime risk of a DFU is as high as 25%. DFUs are characterized by delayed healing and typically require meticulous wound care [227]. Advanced therapies that have shown efficacy in healing DFUs in RCTs include NPWT, placental-derived products, sucrose octasulfate dressings, dermal regeneration templates, and topical oxygen therapy [228,229]. Although most genetic influence on diabetic wound outcomes is mediated through hyperglycemia and vascular disease, there is also evidence for genetic contributions at the level of the wound complication itself. In a GWAS of 699 cases with DFUs and 2695 controls with peripheral neuropathy, the variant rs80028505 in mitogen-activated protein kinase 14 reached genome-wide significance. This genetic signal can be framed as an aspect of genetic susceptibility superimposed on the broader polygenic microvascular burden for diabetes mellitus [230].

Surgical complications are also well documented in diabetic populations and include increased risk for infection, delayed healing, wound dehiscence, seroma, and skin necrosis, likely due to central adiposity and poor perfusion [231,232].

Overall, diabetes produces a pathological environment marked by hyperglycemia, vascular dysfunction, oxidative stress, inflammation, and neuropathy that profoundly disrupts normal tissue repair.

### 4.3. Hypertension

#### 4.3.1. Gene and Protein Function

Genetic risk loci associated with polygenic susceptibility in hypertension do not directly impair wound healing. Instead, they influence pathways that regulate vascular tone, renal sodium handling, endothelial function, and inflammatory signaling. These genetic pathways then coalesce to impair wound healing.

Genetic variants related to various functions of the kidney, including electrolyte and fluid balance, are one group of genes associated with hypertension. Protein variants in the renin-angiotensin aldosterone system (RAAS), such as angiotensinogen (AGT), angiotensin-converting enzyme (ACE), and angiotensin II receptor type I (ATR1), are directly implicated in hypertension as they promote vasoconstriction and sodium retention, which raises blood volume and blood pressure [203]. In terms of endothelial function, *NOS3* and *GUCY1A3* are involved. *NOS3* produces endothelial NO, which drives vasodilation and supports angiogenesis. *GUCY1A3* encodes the α1 subunit of soluble guanylate cyclase (sGC), the NO receptor that generates cyclic-GMP in vascular smooth muscle [204].

Additional genes govern renal sodium transport and vascular smooth muscle excitability. The uromodulin *UMOD* gene modulates NKCC2/ROMK activity in the thick ascending limb, increasing sodium reabsorption and conferring salt-sensitive blood pressure effects [193]. Vascular smooth-muscle calcium handling and excitability are influenced by *ATP2B1* and *CACNB2*. *ATP2B1* encodes a plasma-membrane Ca^2+^-ATPase (PMCA1) in vascular smooth muscle [205]. *CACNB2* encodes a voltage-gated calcium channel β2 subunit influencing membrane excitability and blood pressure [203]. Both genes help control vascular smooth-muscle tone, and variants can shift the balance toward increased vasoconstriction.

Other contributors include *NPR3*, which encodes a natriuretic peptide clearance receptor that influences circulating natriuretic peptide levels, vascular tone and blood volume [203]. Lastly, *SH2B3* encodes LNK, an adaptor that restrains Janus kinase and signal transducer and activator of transcription (JAK–STAT) cytokine signaling. Common variants link inflammation with hypertension and atherosclerosis risk [206]. Taken together, these genes create a biological environment that predisposes individuals to sustained elevations in blood pressure.

Consistent with the highly polygenic architecture of blood pressure regulation, a blood pressure PRS derived from a GWAS of over one million individuals separated high- and low-risk groups with clinically meaningful effect sizes. Top versus bottom deciles differed by 16.9 mm of mercury in systolic blood pressure and had 7.33-fold higher odds of hypertension. Additionally, inclusion of the PRS increased the area under the receiver operating characteristic curve from 0.791 to 0.826 in a hypertension prediction model [233].

#### 4.3.2. Clinical Manifestations

Hypertension is the most prevalent modifiable cardiovascular risk factor globally, affecting nearly 1.4 billion adults globally in 2010, with prevalence continuing to rise [234]. Hypertension is often asymptomatic. Chronic elevation produces target organ damage, such as retinopathy, left ventricular hypertrophy, and nephrosclerosis. Microvascular remodeling raises vascular resistance and impairs tissue perfusion [235].

#### 4.3.3. Wound Healing

Preclinical in vivo studies and clinical evidence support that patients with hypertension experience impaired wound healing, primarily due to microvascular dysfunction, reduced tissue perfusion, and altered vascular remodeling. In vitro studies examining wound healing in the context of hypertension are limited. Additionally, GWAS and PRS analyses examining genetic susceptibility to wound healing impairment in hypertension are currently lacking.

Two major pathogenic mechanisms implicate poor wound healing in hypertension: microvascular rarefaction (reduced capillary density) and endothelial dysfunction (low NO bioavailability). In tandem, both limit nutritive flow to wounds, blunt angiogenesis, and slow keratinocyte and fibroblast responses. A capillary density study found untreated hypertensive individuals to have 10–25% lower skin capillary density than normotensive controls, which mechanistically links perfusion to delayed wound repair [236]. However, an experimental abdominal-wall healing study in rats found that hypertensive rats treated with enalapril had decreased wound-breaking strength on day 7, implying that even with proper hypertensive control, perfusion may still be limited due to low blood flow [237]. Furthermore, in vivo mouse experiments found that NO deficiency impairs vasodilation and angiogenesis and cutaneous healing, implying that NO is important in the wound healing process [238]. Collectively, these data suggest that hypertension disrupts wound healing both structurally and functionally, producing a hypoperfused and hypoxic environment that is not suitable for tissue regeneration.

Multiple cohort and clinical studies demonstrate that hypertension is associated with early and systemic microcirculatory changes, including capillary rarefaction, reduced capillary recruitment, and decreased erythrocyte velocity in skin and other tissues, which compromise oxygen and nutrient delivery essential for wound repair [239,240,241]. These microvascular abnormalities are present in up to 93% of patients with essential hypertension, even before overt organ damage develops [239]. A prospective clinical study found that hypertensive patients undergoing total hip arthroplasty had significantly delayed wound healing compared to normotensive controls. They also had a higher risk of prolonged wound discharge, which increases infection risk [242]. Additionally, hypertension is linked to pathological scarring, such as keloids and hypertrophic scars, likely due to profibrotic changes and chronic inflammation [243].

### 4.4. Obesity

#### 4.4.1. Gene and Protein Function

Obesity is driven by disruptions in the pathways that regulate appetite, energy expenditure, adipocyte differentiation, and thermogenesis. The downstream metabolic and mechanical consequences of obesity, such as chronic inflammation, vascular impairment, and tissue hypoxia, ultimately interfere with proper wound repair.

A large proportion of genes associated with obesity are a part of the leptin-melanocortin axis, the central biological pathway in the brain that controls hunger and satiety. Key genes involved include *LEP*, *LEPR*, *POMC*, *PCSK1*, *MC4R*, *SH2B1*, *BDNF*, *SIM1*, and *ADCY3*. The *LEP* gene encodes leptin, a hormone secreted by adipose tissue that signals satiety, while *LEPR* encodes its hypothalamic receptor. Variants in either can weaken satiety signaling and promote increased food intake. The genes *POMC* and *PCSK1* produce α-melanocyte-stimulating hormone peptides that activate MC4R, the principal hypothalamic receptor controlling appetite suppression. Mutations affecting this pathway reduce melanocortin signaling, driving hyperphagia and weight gain.

Other genes act as modulators of this system. *SH2B1* enhances leptin and insulin signaling, whereas *SIM1* and *BDNF* act as downstream regulators within the melanocortin circuitry, regulating satiety. Additionally, *ADCY3* encodes ADCY3, which localizes to neuronal primary cilia alongside MC4R. Ciliary MC4R–ADCY3 signaling represents a shared pathway underlying many forms of monogenic and syndromic obesity. Together, these genes work to modulate the pathways that control hunger [244].

Adipocyte differentiation and thermogenesis also play a role in obesity. They are modulated by FTO and its downstream targets IRX3 and IRX5. Common regulatory variants within the *FTO* locus rewire an intronic enhancer to upregulate IRX3 and IRX5, promoting the development of energy-storing white adipocytes [245]. This shift promotes reduced energy expenditure and increased fat accumulation. Together, these genetic variants produce a biological environment that favors excess adiposity and metabolic dysfunction that is detrimental to wound healing.

Recent multi-ancestry PRS demonstrate that diffuse genetic susceptibility can translate into large differences in obesity risk. A multi-ancestry PRS for BMI explained 17.6% of BMI variation among United Kingdom Biobank participants of European ancestry. In European-like ancestry groups, obesity prevalence in the top one percent of the score distribution was 69.5%, compared with 1.7% in the bottom one percent. Across populations, each standard deviation increase in the score was associated with an approximately 1.9-fold to 2.6-fold increase in odds of obesity [246].

#### 4.4.2. Clinical Manifestations

Obesity affects over 878 million [247,248]. It is a chronic, multifactorial, and polygenic metabolic disorder, characterized by excessive adiposity and systemic metabolic dysregulation. It is associated with insulin resistance, dyslipidemia, and hypertension. Lymphedema risk also rises with severe obesity [249]. In obesity, cutaneous and mechanical complications are frequent. Obese individuals often have intertriginous dermatitis, pressure injuries, and venous stasis ulcers due to impaired lymphatic drainage and increased venous pressure [250].

#### 4.4.3. Wound Healing

It is well known that obesity is one of the most prevalent risk factors for chronic wounds and postoperative complications. The genetic variants associated with obesity do not directly impact wound repair. Instead, preclinical data shows that the obesity phenotype, marked by chronic inflammation, impaired angiogenesis, reduced perfusion, and mechanical skin stress, creates an environment hostile to normal healing. Strong GWAS and PRS analyses specifically focused on wound healing outcomes in obesity are not available.

Experimental in vivo mouse studies found that obesity induces a state of low-grade systemic inflammation characterized by M1-skewed macrophage activation and persistent release of pro-inflammatory cytokines [251]. These cytokines delay the transition from inflammation to cell proliferation in wounds. Simultaneously, analysis of patient serum shows that levels of adiponectin, which is an anti-inflammatory pro-angiogenic adipokine, are reduced in obese individuals. This further impairs keratinization, migration, and endothelial repair [252]. Adipose tissue expansion also leads to perfusion deficits and reduced capillary density, as seen when comparing obese and non-obese patients during laparoscopy. This limits oxygen diffusion and results in tissue hypoxia [253]. Mechanical factors like friction, shear, and moisture also accumulate in skin folds, which promotes epidermal maceration and microtrauma [208].

Large cohort studies show that obese patients have increased postoperative wound healing complications. A prospective cohort of 307 postoperative patients showed that each 5 kg/m^2^ increase in BMI correlated with a 13% risk of surgical skin infection [254]. In bariatric-surgery incisions, wounds in obese subjects exhibited impaired neovascularization and reduced tensile strength compared to matched lean patients, confirming physiological wound healing impairment [249]. A systematic review and meta-analysis of over 400,000 patients with lower limb trauma found that obesity significantly increased the risk of wound complications and prolonged hospital stays compared to non-obese patients [255].

### 4.5. Peripheral Artery Disease

#### 4.5.1. Gene and Protein Function

In peripheral artery disease (PAD), several genetic risk loci are associated with polygenic susceptibility. These genes influence lipid metabolism, inflammation, vascular remodeling, and thrombosis, which further disrupts tissue repair.

Many PAD-associated genes regulate low-density lipoprotein (LDL) metabolism and influence the formation of atherosclerotic plaque. Variants in *LDLR*, *APOB*, *PCSK9*, *SORT1*, and *LPA* modulate LDL levels and vascular deposition, which contribute to plaque formation. *LDLR* encodes the LDL receptor, which binds apolipoprotein B-100 and apolipoprotein E on circulating lipoproteins and mediates their endocytosis into hepatocytes for cholesterol clearance. *APOB* encodes apolipoprotein B-100, the structural protein of very low-density lipoprotein (VLDL), intermediate-density lipoprotein (IDL), and LDL. It serves as the ligand that allows these lipoproteins to bind LDL receptors. *PCSK9* encodes a protein that binds LDL receptors and targets them for lysosomal degradation, thereby regulating the number of receptors available to clear LDL cholesterol. Gain-of-function variants in *PCSK9* diminish receptor availability and raise LDL cholesterol. *SORT1* encodes sortilin-1, a sorting receptor in hepatocytes that directs secretion and trafficking of apolipoprotein B-containing lipoproteins, influencing LDL metabolism [207]. Finally, *LPA* encodes apolipoprotein(a), which covalently attaches to apolipoprotein B-100 to form lipoprotein(a). It structurally resembles plasminogen and carries oxidized phospholipids. Elevated apolipoprotein(a) is one of the strongest inherited PAD risk factors [207].

Inflammatory and vascular remodeling genes also contribute. *IL6*, *CXCL12*, and *SH2B3* modulate systemic inflammation, leukocyte recruitment, and vascular remodeling. *IL6* encodes IL-6, a cytokine that drives the acute-phase response, stimulates hepatic C-reactive protein and fibrinogen production, and shapes immune cell differentiation. *CXCL12* encodes stromal cell–derived factor 1, a chemokine that recruits stem and progenitor cells as well as leukocytes to sites of ischemia or injury, supporting repair and angiogenesis. *SH2B3* encodes LNK, an adaptor protein that negatively regulates JAK-STAT signaling, thereby limiting cytokine and growth factor responses in hematopoietic and vascular cells [207].

Genes in the coagulation pathway, such as *F2* and *F5* variants, increase thrombosis risk, which can accelerate ischemia by promoting vascular occlusion. *F2* encodes prothrombin, the precursor of thrombin, which cleaves fibrinogen to fibrin and activates platelets and other clotting factors. *F5* encodes coagulation factor V, a cofactor in the prothrombinase complex that accelerates the conversion of prothrombin to thrombin [207].

Additional susceptibility arises from variants in the 9p21 locus of *CDKN2B-AS1/ANRIL*, which encodes a long noncoding RNA that regulates CDKN2A and CDKN2B, controlling cell cycle progression and proliferation of vascular smooth muscle cells. It also influences plaque stability [207]. Structural integrity of blood vessels and the stability of the plaque are further shaped by *COL4A1* and *COL4A2*, which encode the α1 and α2 chains of type IV collagen, the fundamental scaffold of basement membranes [207].

#### 4.5.2. Clinical Manifestations

PAD affects more than 200 million people worldwide [256,257]. Early features include intermittent claudication, reduced pulses, and femoral or popliteal artery bruits. As disease progresses, patients may experience pain at rest, thickened toenails, and trophic skin changes, such as thin, shiny, hairless skin. Complications include non-healing wounds and acute limb ischemia [258].

#### 4.5.3. Wound Healing

Wound healing impairment and production of PAD-associated ulcers are well documented in the preclinical and clinical literature and arise directly from chronic limb ischemia.

Narrowed arteries limit perfusion and lower tissue oxygen tension below the threshold required for fibroblast proliferation, keratinocyte migration, and angiogenesis. Without oxygen, cells rely on glycolysis, producing insufficient ATP for repair and weakening collagen hydroxylation, which reduces tensile strength of granulation tissue, as shown in a rat model [259]. In vitro studies and in vivo rat models have also shown that endothelial dysfunction further compounds this by reducing NO bioavailability, blunting vasodilation, and impairing endothelial cell proliferation [260]. Hypoxia would normally stabilize HIF-1α and drive VEGF-mediated angiogenesis. However, a mouse model has shown that in PAD, persistent oxidative stress destabilizes HIF-1α and degrades VEGF signaling, leading to poor collateral vessel growth [261]. At the inflammatory level, ischemic tissue accumulates ROS that keep macrophages in a pro-inflammatory (M1) state and prolong neutrophil infiltration. This results in excess MMP activity, which degrades ECM faster than fibroblasts can rebuild it, leaving the wound in the inflammatory phase [262].

A cross-sectional case–control study showed that patients with PAD had significantly higher levels of MMPs compared to controls and that MMP level correlated with disease severity [263]. A clinical retrospective study found that poor perfusion reduces delivery of immune cells and antibiotics, increasing vulnerability to colonization, biofilm formation, and osteomyelitis in patients with diabetic foot infections and concomitant symptomatic PAD [264]. Basement membrane disruption and microvascular rarefaction exacerbate these effects, reducing capillary density and impairing leukocyte transmigration, as seen in a clinical cross-section study comparing calf muscles from patients with PAD and those without [265].

Polygenic stratification is now being extended beyond PAD diagnosis to limb outcomes that closely relate to nonhealing wounds and amputation risk. In a cohort study evaluating PRS for PAD, each standard deviation increase in the PRS was associated with an odds ratio of 1.63 for PAD, and the top twenty percent of the score remained associated with an odds ratio of 1.68 after adjustment for clinical risk factors. Among individuals with established PAD, high PRS status was associated with higher risk of incident major adverse limb events (including major amputation and acute limb ischemia), with hazard ratios ranging from 1.56 to 1.75 across three cohorts. This provides a direct genetic-to-clinical link from inherited atherosclerotic susceptibility to PAD and limb outcomes [266].

## 5. Conclusions

**Figure 2:**
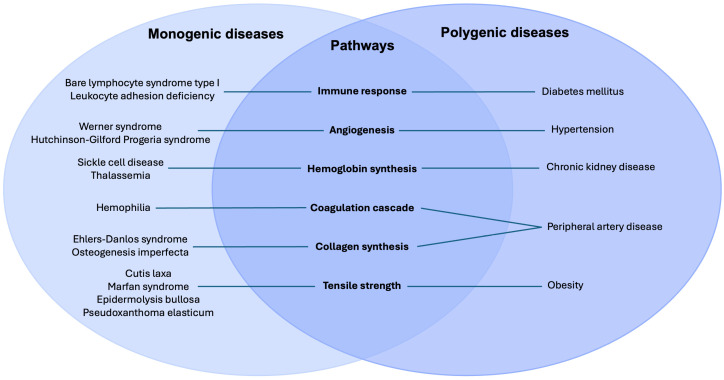
Simplified diagram of monogenic and polygenic diseases that affect key pathways in wound healing. Note that some diseases may affect more pathways, as described in the main text.

This review has charted the profound impact of genetic architecture on wound healing, demonstrating that repair vulnerability is not a binary state but a continuum (see Figure 2). This spectrum spans from catastrophic, single-pathway failures in monogenic disorders to the systemic, multifactorial dysregulation characteristic of polygenic diseases. In the case of monogenic conditions, such as LAD, we find that mutations in a single gene are sufficient to derail a specific, critical component of repair, such as inflammatory cell trafficking. These rare disorders, while devastating, serve as “human knockouts” that provide evidence for the essentiality of these pathways.

The true clinical burden of impaired healing, however, lies in high-prevalence polygenic conditions. This review has shown how diseases like DM, PAD, and obesity create a “non-permissive” healing environment through a convergence of multiple, subtly dysregulated pathways. The mechanistic insights gleaned from monogenic disorders serve as a critical lens through which to understand these more complex pathologies. For example, the frank structural disarray of the ECM in cutis laxa provides a model for understanding the more nuanced, but equally detrimental, fibrotic and disorganized ECM remodeling seen in chronic diabetic ulcers. Similarly, the overt immune cell dysfunction of LAD provides a framework for interpreting the state of “paralyzed” inflammation in ischemic or venous ulcers. Thus, the central finding of this is that while genetic causes differ, the mechanistic failure—ischemic, inflammatory, or structural—often converges on the same final common pathways.

**Figure 3:**
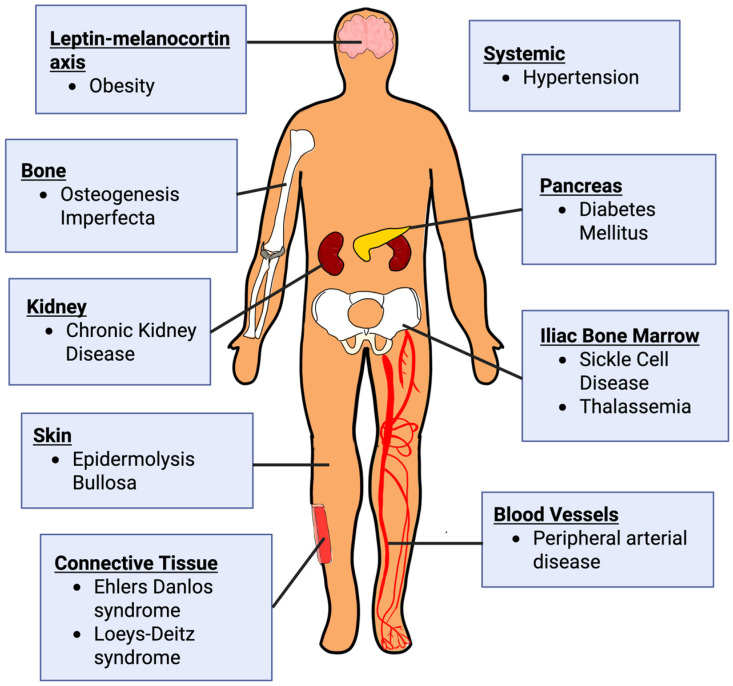
Systems, organs, tissues, and systems affected by monogenic and polygenic diseases. Monogenic conditions shown include osteogenesis imperfecta, Ehlers–Danlos syndrome, epidermolysis bullosa, Loeys–Dietz syndrome, sickle cell disease, and thalassemia. Polygenic conditions depicted include obesity, hypertension, diabetes mellitus, chronic kidney disease, and peripheral arterial disease. Created in BioRender. Mueller, S. (2025) https://BioRender.com/fq133t0 (accessed 21 November 2025).

The synthesis of these findings has profound implications for clinical practice. It argues for a fundamental shift away from a “one-size-fits-all” wound care paradigm and toward a more mechanistically driven, personalized approach. Crucially, this approach recognizes that impediments to wound healing do not stem from a single source but can originate in distinct biological compartments—ranging from the structural integrity of the extracellular matrix to the competency of the immune or vascular systems (see Figure 3). Understanding a patient’s underlying genetic predisposition—whether it be a known monogenic diagnosis or a polygenic risk profile for DM and PAD—is not merely academic. It directly informs clinical suspicion and management. A wound in a patient with cEDS demands aggressive mechanical offloading and advanced suturing techniques, whereas a wound in a patient with LAD may prioritize aggressive antimicrobial and immune-supportive therapies. For the vast majority of patients with polygenic disease, our findings underscore the necessity of treating systemic disease as the primary wound care strategy, rather than focusing exclusively on the local wound bed.

This review also highlights critical gaps in our knowledge. While the link between monogenic disorders and their specific wound healing failures is sometimes clear, the precise genetic variants and gene-environment interactions that drive non-healing in polygenic disease remain to be fully elucidated. Future research must leverage multi-omics approaches (genomics, proteomics, metabolomics) on human chronic wound tissue to identify the specific molecular signatures of recalcitrance. This will be essential for developing novel “pathway-specific” therapeutics that can be matched to a patient’s underlying pathology. Furthermore, prospective clinical trials in wound healing must begin to stratify patients based on these genetic and molecular profiles to identify which therapies work for which patients, ultimately ushering in an era of precision wound care.

## Figures and Tables

**Table 1:** Summary of monogenic disorders associated with wound healing impairment—grouped by hematological diseases and primary immunodeficiencies, premature aging disorders, and connective-tissue disorders—listing the primary gene(s), wild-type protein function, and the strength of evidence for impaired healing. Diseases are listed in alphabetical order within each group. Strength of evidence reflects the preponderance and quality of preclinical and clinical data. Abbreviations: TAP, transporter associated with antigen processing; MHC, major histocompatibility complex; FVIII, factor VIII; FIX, factor IX; HbA, adult hemoglobin; TGF-β, transforming growth factor-β; ATP, adenosine triphosphate; ECM, extracellular matrix.

Disorder	Gene	Wild-Type Protein Function	Strengths and Limitations of Evidence for Wound Healing Impairment
Hematological Diseases and Primary Immunodeficiencies			
Bare Lymphocyte Syndrome Type I [15,16]	*TAP1/2*	TAP complex; required for peptide expression on MHC I class proteins	Limited in vitro and in vivo studies Clinical studies limited to case reports
Hemophilia	*F8*	FVIII, FIX; components of the intrinsic pathway, necessary for clot formation	Strong in vitro, in vivo, and clinical studies
	*F9*		
Leukocyte Adhesion Deficiency [17]	*ITGB2*	CD18; essential for firm adhesion, chemotaxis, and transmigration of neutrophils across the endothelium	
	*SLC35C1*	GDP-fucose transporter; produces selectin ligands required for leukocyte rolling and endothelial adhesion.	Strong in vitro, in vivo, and clinical studies
	*FERMT3*	Kindlin-3; enables effective leukocyte adhesion, platelet aggregation, and immune cell trafficking	
Sickle Cell Disease [18,19]	*HBB*	Beta-globin chain of HbA; required for oxygen transport and RBC stability	Strong in vitro, in vivo, and clinical studies
Thalassemia [20,21,22,23]	*HBB*	See Sickle Cell Disease	Limited in vitro and in vivo Strong clinical studies
	*HBA1/2*	Alpha-globin chains of HbA; essential for hemoglobin assembly and oxygen delivery	
Aging-Related Syndromes			
Ataxia Telangiectasia [24]	*ATM*	Kinase; coordinates the DNA double-strand break response and cell-cycle checkpoints	Strong in vitro and in vivo studies Clinical studies limited to case reports
Hutchinson-Gilford Progeria Syndrome [25]	*LMNA*	Lamin A/C; maintains nuclear structure and regulates chromatin and gene expression	Limited in vitro studies, moderate in vivo studies No clinical studies
Werner Syndrome [26]	*WRN*	RecQ helicase/exonuclease; supports DNA replication/repair and telomere maintenance	Strong in vitro, in vivo, and clinical studies
Connective Tissue Diseases			
Cutis Laxa [27]	*ELN*	Core elastin protein; provides tissue elasticity	Moderate in vitro and in vivo studies Clinical studies limited to case reports
	*FBLN5*, *FBLN4/EFEMP2*	Scaffold proteins for elastin crosslinking and deposition	
	*LTBP4*	TGF-β binding protein; regulates elastic fiber maturation	
	*ATP6V0A2*	H+ ATPase; maintains Golgi body acidification for proper ECM protein processing	
Ehlers-Danlos Syndrome [28]	*COL5A1/2*	Type V collagen; provides support to the dermis, tendons, and muscles; critical in Type I collagen fibrillogenesis	Strong in vitro, in vivo, and clinical studies
	*COL3A1*	Type III collagen; provides tensile strength in the tunica media of blood vessels and hollow organs	
Epidermolysis Bullosa [29,30,31,32,33]	*KRT5/14*	Basal keratin intermediate filaments; provide structural support to basal keratinocytes	Strong in vitro, in vivo, and clinical studies
	*PLEC*, *DST*	Plakin cytolinker proteins; anchor keratin intermediate filaments to hemidesmosomes	
	*CD151*	Tetraspanin; organizes laminin-binding integrins	
	*EXPH5*	Rab27 effector; involved in vesicle trafficking, keratinocyte adhesion, membrane repair, and barrier maintenance	
	*COL7A1*	Type VII collagen; component of anchoring fibrils that secure the basement membrane to dermal collagen	
	*LAMA3*, *LAMB3*, *LAMC2*	Components of laminin-332; integrin that mediates keratinocyte adhesion, polarity, and migration	
	*ITGA6*, *ITGB4*, *ITGA3*	Laminin-binding integrin subunits, critical for hemidesmosome formation, cell–matrix adhesion, and signaling during re-epithelialization	
	*FERMT1*	Kindlin-1; keratinocyte stability, integrin activation	
Loeys-Dietz Syndrome [34]	*TGFBR1/2*, *TGFB2/3*	TGF-β ligand and receptors	Strong in vitro and in vivo studies Clinical studies limited to case reports and case series
	*SMAD2/3*	Intracellular mediators of TGF-β signaling; regulates inflammation and fibroblast activation	
Marfan Syndrome [35,36]	*FBN1*	Structural glycoprotein forming microfibrils; provides elasticity and regulates TGF-β bioavailability in connective tissue	Moderate in vitro and in vivo studies Clinical evidence limited to case reports
Osteogenesis Imperfecta [37]	*COL1A1/2*	Type I collagen; provides tensile strength in bone, skin, and soft tissue	Limited in vitro studies Strong in vivo and clinical studies
Pseudoxanthoma Elasticum [38,39]	*ABCC6*	ATP-binding cassette transporter; prevents ectopic calcification of elastic fibers in skin and vasculature	Strong in vitro studies Limited in vivo studies Clinical evidence limited to case reports

**Table 2:** Summary of polygenic diseases associated with wound healing impairment listing the primary gene(s), wild-type protein function, and the strength of evidence for impaired healing. Diseases are listed in alphabetical order within each group. Strength of evidence reflects the preponderance and quality of preclinical and clinical data. Abbreviations: GMB, glomerular basement membrane; TF, transcription factor; GWAS, genome-wide association study; PRS, polygenic risk score; GLP-1, glucagon-like peptide-1; AGT, angiotensinogen; ACE, angiotensin-converting enzyme; ATR1, angiotensin II receptor type I; NO, nitric oxide; sGC, soluble guanylate cyclase; JAK-STAT, Janus kinase and signal transducer and activator of transcription; LDL, low-density lipoprotein; IL, interleukin.

Disease	Gene	Wild-Type Protein Function	Strengths and Limitations of Evidence for Wound Healing Impairment
Chronic Kidney Disease [193,194,195,196]	*UMOD*	Uromodulin; modulates NKCC2/ROMK transporters	Strong in vitro, in vivo, and clinical studies Limited GWAS/PRS studies
	*PKD1/2*	Polycystin 1/2; regulate calcium signaling	
	*COL4A3/4/5*	Type IV collagen, GBM stability	
	*HNF1B*	TF; nephrogenesis	
Diabetes Mellitus [197,198,199,200,201,202]	*DR3/4*, *DQ2*/*8*	Antigen-presenting molecules on immune cells	Strong in vitro, in vivo, and clinical studies Strong GWAS/PRS studies
	*PTPN22*	lymphoid-specific tyrosine phosphatase; negative regulator of T-cells	
	*CTLA4*	T-cell surface protein; immune checkpoint regulator	
	*TCF7L2*	TF; regulates GLP-1 production and insulin secretion	
	*SLC20A8*	Zinc transporter; insulin storage	
	*KCNJ11*	Kir6.2; regulates insulin secretion	
	*PPARG*	PPAR-γ; adipocyte differentiation and lipid metabolism	
	*FTO*	N^6^-methyladenosine demethylase; energy homeostasis and appetite control	
Hypertension [193,203,204,205,206]	*AGT*	AGT; precursor to angiotensin	Limited in vitro studies Strong in vivo and clinical studies Limited GWAS/PRS studies
	*ACE*	ACE; converts angiotensin I to angiotensin II	
	*AGTR1*	ATR1; regulates sodium reabsorption and vasoconstriction	
	*NOS3*	NO; causes vasodilation	
	*GUCY1A3*	sGC; involved in vasodilation	
	*ATP2B1*	Ca^2+^-ATPase; involved in vasodilation	
	*CACNB2*	Ca^2^ channel; influences vascular smooth muscle membrane excitability	
	*NPR3*	Natriuretic peptide clearance receptor; regulates blood volume	
	*SH2B3*	LNK; involved in JAK-STAT cytokine signaling	
Obesity	*LEP*, *LEPR*	Leptin, leptin receptor; regulate satiety and energy expenditure	Strong in vitro, in vivo, and clinical studies Limited GWAS/PRS studies
	*POMC*, *PCSK1*	α-melanocyte-stimulating hormone peptides; control appetite suppression	
	*SH2B1*	Mediator; enhances leptin and insulin signaling	
	*SIM1*, *BDNF*, *ADCY3*	TF, neurotransmitter, mediator; regulate satiety	
	*FTO*	N^6^-methyladenosine demethylase, involved in adipocyte differentiation	
Peripheral Artery Disease [207]	*LDLR*, *APOB*, *PCSK9*, *SORT1*, *LPA*	LDL receptor, apolipoprotein B-100, LDL receptor ligan, sortilin-1, apolipoprotein(a); involved in LDL metabolism and vascular deposition	Strong in vitro, in vivo, and clinical studies Moderate GWAS/PRS studies
	*IL6*, *CXCL12*, *SH2B3*	IL-6, stromal cell-derived factor 1, LNK; modulate systemic inflammation, leukocyte recruitment, vascular remodeling	
	*F2*, *F5*	Prothrombin, Factor V; involved in coagulation cascade	
	*CDKN2B-AS1/ANRIL*	Long non-coding RNA; controls proliferation of vascular smooth muscle cells, contributes to plaque stability	
	*COL4A1/2*	Type IV collagen; provides structural integrity to blood vessels	

## Data Availability

No new data were created or analyzed in this study.

## Abbreviations

The following abbreviations are used in this manuscript and are listed in alphabetical order:

A-T	ataxia telangiectasia
ACE	angiotensin-converting enzyme
AGE	advanced glycation end product
AGT	angiotensinogen
ATR1	angiotensin II receptor type I
BHFS	Hemoglobin Bart’s hydrops fetalis syndrome
BLS I	bare lymphocyte syndrome type I
BMI	body mass index
cEDS	Classical Ehlers-Danlos syndrome
CKD	chronic kidney disease
DEB	dystrophic epidermolysis bullosa
DFU	diabetic foot ulcer
DM	diabetes mellitus
EB	epidermolysis bullosa
EBS	epidermolysis bullosa simplex
ECM	extracellular matrix
EDS	Ehlers-Danlos syndrome
EGF	epidermal growth factor
EGFR	epidermal growth factor receptor
EPC	endothelial progenitor cell
ER	endoplasmic reticulum
FGF	fibroblast growth factor
FIX	factor IX
FVIII	factor VIII
GBM	glomerular basement membrane
GDP	guanosine diphosphate
GLP-1	glucagon-like peptide-1
GWAS	genome-wide association study
HbA	hemoglobin A
HbH	hemoglobin H
HbS	hemoglobin S
hEDS	Hypermobile Ehlers-Danlos syndrome
HGF	hepatocyte growth factor
HGPS	Hutchinson-Gilford progeria syndrome
HIF-1α	hypoxia-inducible factor 1-α
HLA	human leukocyte antigen
HSCT	hematopoietic stem cell transplantation
IDL	intermediate-density lipoprotein
IL	interleukin
iPSC	induced pluripotent stem cell
JAK-STAT	Janus kinase and signal transducer and activator of transcription
JEB	junctional epidermolysis bullosa
JNK	c-Jun N-terminal kinase
KEB	Kindler epidermolysis bullosa
LAD	leukocyte adhesion deficiency
LAD-I	Type I LAD
LAD-II	Type II LAD
LAD-III	Type III LAD
LDL	low-density lipoprotein
LDS	Loeys-Dietz syndrome
LU	leg ulcer
MAPK	mitogen-activated protein kinase
MFS	Marfan syndrome
MHC	major histocompatibility complex
MIAMI	marrow-isolated adult multilineage inducible
MMP	matrix metalloproteinase
MSC	mesenchymal stromal cell
NAD+	nicotinamide adenine dinucleotide
NK cells	natural killer cells
NO	nitric oxide
NPWT	negative pressure wound therapy
OI	osteogenesis imperfecta
PAD	peripheral artery disease
PI3K-Akt	phosphatidylinositol 3-kinase-protein kinase B
PPAR-γ	peroxisome proliferator–activated receptor gamma
PRS	polygenic risk score
PDGF	platelet growth factor
PXE	pseudoxanthoma elasticum
RAAS	renin-angiotensin-aldosterone system
RCT	randomized controlled trial
SCC	squamous cell carcinoma
SCD	sickle cell disease
sGC	soluble guanylate cyclase
T1DM	type I diabetes mellitus
T2DM	type 2 diabetes mellitus
TAP	transporter associated with antigen processing
TGF-β	transforming growth factor-β
TIMP	tissue inhibitors of metalloproteinase
TNF-α	tumor necrosis factor-α
TNF-β	tumor necrosis factor-β
vEDS	Vascular Ehlers-Danlos syndrome
VEGF	vascular endothelial growth factor
VLDL	very low-density lipoprotein
WS	Werner syndrome
